# Cu(II) and Cd(II) Removal Efficiency of Microbially Redox-Activated Magnetite Nanoparticles

**DOI:** 10.1021/acsearthspacechem.2c00394

**Published:** 2023-10-09

**Authors:** Timm Bayer, Ran Wei, Andreas Kappler, James M. Byrne

**Affiliations:** † Geomicrobiology Group, Department of Geoscience, University of Tuebingen, Schnarrenbergstrasse 94-96, 72076 Tuebingen, Germany; ‡ Environmental Systems Analysis, Department of Geoscience, University of Tuebingen, Schnarrenbergstrasse 94-96, 72076 Tuebingen, Germany; § Cluster of Excellence: EXC 2124: Controlling Microbes to Fight Infection, 72074 Tuebingen, Germany; ∥ School of Earth Sciences, University of Bristol, Wills Memorial Building, Queens Road, BS8 1RJ Bristol, United Kingdom

**Keywords:** heavy metals, adsorption, magnetite, nanoparticles, iron oxidation, iron reduction

## Abstract

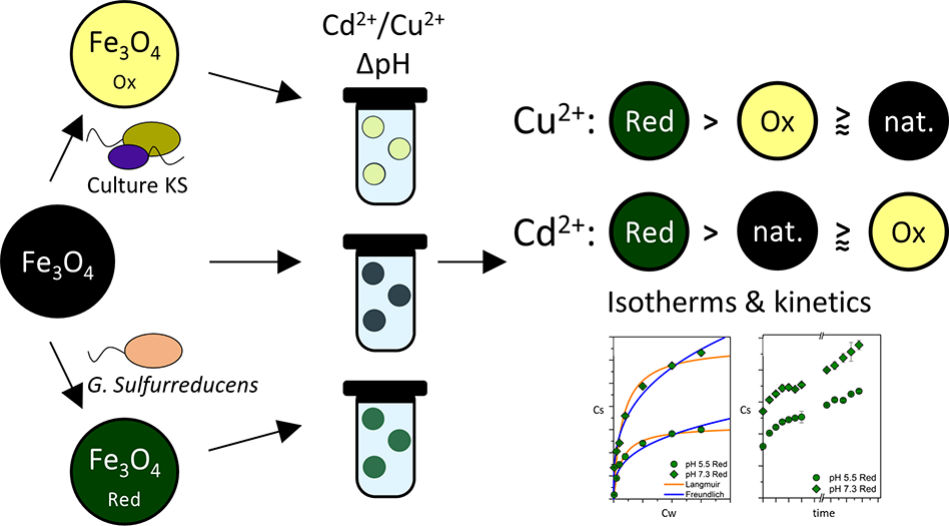

Heavy metal pollutants in the environment are of global concern due to their risk of contaminating drinking water and food supplies. Removal of these metals can be achieved by adsorption to mixed-valent magnetite nanoparticles (MNPs) due to their high surface area, reactivity, and ability for magnetic recovery. The adsorption capacity and overall efficiency of MNPs are influenced by redox state as well as surface charge, the latter of which is directly related to solution pH. However, the influence of microbial redox cycling of iron (Fe) in magnetite alongside the change of pH on the metal adsorption process by MNPs remains an open question. Here we investigated adsorption of Cd^2+^ and Cu^2+^ by MNPs at different pH values that were modified by microbial Fe(II) oxidation or Fe(III) reduction. We found that the maximum adsorption capacity increased with pH for Cd^2+^ from 256 μmol/g Fe at pH 5.0 to 478 μmol/g Fe at pH 7.3 and for Cu^2+^ from 229 μmol/g Fe at pH 5.0 to 274 μmol/g Fe at pH 5.5. Microbially reduced MNPs exhibited the greatest adsorption for both Cu^2+^ and Cd^2+^ (632 μmol/g Fe at pH 7.3 for Cd^2+^ and 530 μmol/g Fe at pH 5.5 for Cu^2+^). Magnetite oxidation also enhanced adsorption of Cu^2+^ but inhibited Cd^2+^. Our results show that microbial modification of MNPs has an important impact on the (im-)mobilization of aqueous contaminations like Cu^2+^ and Cd^2+^ and that a change in stoichiometry of the MNPs can have a greater influence than a change of pH.

## Introduction

Advancements in industrialization and agriculture have led to increasing heavy metal concentrations in the environment, causing concerns about drinking water quality.^1^ Widespread use of cadmium (Cd) in industrial processes such as battery manufacturing and of copper (Cu) in plumbing resulted in increased concentrations of these contaminants in the environment.^2,3^ Removal of these contaminants to achieve safe drinking water and maintain fertile soil is of high interest and continuous investigation.^1,4^ Prolonged ingestion of increased concentrations of heavy metals can lead to adverse effects. Cd is a heavy metal without known metabolic function and is toxic even in very low concentrations.^5^ In addition to battery manufacturing and combustion, Cd is widespread as a contaminant in agricultural phosphorus-based fertilizers.^6,7^ Cd is considered carcinogenic, and prolonged exposure to Cd can lead to kidney diseases.^5^ In contrast to Cd, Cu is an essential trace metal, but high concentrations have been associated with liver damage and possibly gastrointestinal diseases in humans.^8^ High Cu concentrations cause oxidative stress through reactive oxygen species on a molecular level.^9^ Cu is introduced into the environment through industry and in vineyards and orchards where it is used as a fungicide.^10^ Cu and Cd are not biodegradable, accumulate in the environment, and ultimately end up in bodies of water.

A range of techniques such as membrane filtration and ion exchange are used to treat heavy metal pollutions.^11^ Adsorption is a frequently used method for heavy metal removal due to the relative simplicity of implementation and economic efficiency.^12,13^ Iron(III) (Fe(III)) (oxyhydr)oxides are commonly used as adsorbents to remove contaminants from solution and are used commercially.^14^ In Vietnam the precipitation of Fe(III) (oxyhydr)oxides in household sand filters has been shown to be highly effective at removing dissolved toxic arsenic (As).^15,16^ Fe oxides generally have a high surface area and reactivity, which makes them an ideal adsorbent material.^17^ Magnetite is a naturally occurring mixed-valent Fe oxide that contains both Fe(II) and Fe(III) (Fe(III)_2_Fe(II)O_4_). It can be formed abiotically through weathering^18^ and biologically through dissimilatory Fe(III) reduction^19^ and oxidation.^20,21^ Magnetite nanoparticles (MNPs) especially can be applied in heavy metal remediation since they have high specific surface area, redox reactivity and can be magnetically extracted. Recent studies investigated the adsorption of chromium (Cr) and As by bioengineered magnetite^22^ and the removal of Cr by magnetite-coated sand.^23,24^ Due to its multivalent nature, unlike most other iron oxides, magnetite can be both oxidized and reduced via microbial activity of Fe(II)-oxidizing and Fe(III)-reducing bacteria, respectively. This was previously shown for the photoautotrophic Fe(II)-oxidizing bacteria *Rhodopseudomonas palustris* TIE-1 and Fe(III)-reducing bacteria *Geobacter sulfurreducens*.^25^ Changing the Fe(II)/Fe(III) ratio in magnetite can ultimately lead to its dissolution through reductive dissolution or transformation to maghemite (maghemitization) through oxidation.^26,27^ However, magnetite can have a wide range of Fe(II)/Fe(III) ratios while not undergoing transformation to a different mineral and maintaining the crystal structure of magnetite.^25,28^ The change of the stoichiometry in MNPs can greatly improve the remediation capacity of magnetite, which was previously shown for Cr.^29,30^ Conversely, it has also been shown that microbial activity decreased the reactivity of MNPs toward As(V)^22^ and that magnetite surface passivation can occur through chromium reduction to Cr(III), resulting in a surface layer maghemitization.^31^ Studies have shown that increase of Fe^2+^ led to greater reduction of nitroaromatic compounds^32^ and that an increased stoichiometry in magnetite enhanced the capacity to bind antibiotics.^33^ Additionally, the recharging of magnetite with Fe^2+^ for increased reactivity has been demonstrated.^34^ Previous research investigating removal of Cu^2+^ with magnetite mainly focused on the adsorption process without accounting for the Fe(II)/Fe(III) ratio of magnetite or modified particles with magnetite to obtain magnetic removal.^35,36^ The stoichiometry however directly influences the surface properties of MNPs which are also a consequence of the pH value of the solution.

In this study we consider the impact of microbially mediated redox reactions on the reactivity of MNPs toward two divalent heavy metals. In particular we oxidized MNPs by the autotrophic nitrate-reducing Fe(II)-oxidizing culture KS,^37,38^ reduced magnetite by the Fe(III)-reducing bacterium *G. sulfurreducens*, and compared the adsorption of Cu^2+^ and Cd^2+^ against unaltered (native) MNPs. We also tested how changes in pH influence adsorption to the three types of MNPs. The results presented below consider both adsorption isotherms and kinetic experiments of Cd^2+^ and Cu^2+^ on oxidized mag_ox_, reduced mag_red_, and native mag_nat_ MNPs.

## Materials and Methods

### Safety Statement

No unexpected or unusually high safety hazards were encountered during experiments performed for this research.

### Preparation of Solutions

For all adsorption experiments, anoxic stock solutions of the adsorbent (mag_ox_, mag_red_, or mag_nat_), adsorbate (CuNO_3_ or CdNO_3_), and solvent (0.1 M NaNO_3_) were adjusted to the desired pH 2 days prior to the start of the experiment. pH was adjusted with diluted puriss.HNO_3_ and NaOH. The pH was checked at least twice per day and corrected accordingly. All solutions were prepared with ultrapure H_2_O (Milli-Q, Merck Milli-pore). Glassware and rubber stoppers were soaked for 10 min with 1 M HCl and then rinsed 3 times with Milli-Q-H_2_O.

### Magnetite Synthesis, Oxidation, Reduction, and Stoichiometry

Magnetite was produced according to Pearce et. al^39^ but modified to allow magnetite synthesis outside of the glovebox and on a larger scale. For oxidation, magnetite was incubated with the autotrophic nitrate-reducing iron-oxidizing culture KS as previously described^40^ with 4 mM NaNO_3_ for 7 days, with an increased inoculum of 10% v/v. We previously detected that culture KS can oxidize magnetite. For reduction, magnetite was incubated with 10% v/v of iron-reducing *G. sulfurreducens* with 20 mM sodium acetate for 5 days.^25^ After incubation magnetite was washed at least 5 times with 0.1 M NaNO_3_ to remove all cells, and minerals were collected with a strong bar magnet after each washing step. Magnetite stoichiometry was measured by the ferrozine assay^41^ adapted to microtiter plates. Removal of biomass was checked by measuring DOC (dissolved organic carbon) (High TOC II, Elementar, Elementar Analysensysteme GmbH, Germany) of a washed sample and via fluorescence microscopy by applying a dead/live stain (BacLight Bacterial Viability Kits, Molecular Probes) to screen for any leftover cells after the washing procedure.

### Adsorption Isotherms

All experiments were set up in an anoxic glovebox. Triplicate bottles of increasing concentrations of Cu^2+^ or Cd^2+^ and controls (no MNPs/no adsorbate) were prepared by adding anoxic stock solutions of NaNO_3_ followed by well-mixed MNPs and then Cu^2+^/Cd^2+^ to obtain a total volume of 5 mL in each bottle. The final concentration of magnetite was 9 mM (as total Fe). Concentration of adsorbate depended on the conducted experiment. The bottles were sealed with rubber stoppers, mixed, and then incubated in the dark at 25 °C on a rolling shaker. After 24 h of incubation the bottles were sampled in the glovebox. Two milliliters was removed with a pipet and centrifuged for 2 min at 10 000*g*, and the sample then was split into pellet and supernatant fractions. Outside of the glovebox, the pellet was dissolved in 2 mL of 6 M puriss.HCl for 15 min. Supernatant and dissolved pellet were diluted in 2% puriss.HNO_3_ and measured with microwave plasma-atomic excitation spectroscopy (Agilent 4200 MP-AES, Agilent Technologies). In total, 12 isotherms were obtained for the following: Cd^2+^ + mag_nat_ at pH 5.0, pH 5.5, 6.5, and 7.3; Cd^2+^ + mag_ox_ and Cd^2+^ + mag_red_ at pH 5.5 and 7.3; Cu^2+^ + mag_nat_ at pH 5.0 and 5.5; Cu^2^ + mag_ox_ and Cu^2+^ + mag_red_ at pH 5.5. Experiments with Cu^2+^ were only conducted at pH 5.0 and 5.5. The pH was chosen to avoid precipitation of Cu(OH)_2_ which occurs for concentrations of 2 mM (as present in starting stock solutions) above pH 5.53, with the solubility product of Cu-hydroxide being *K*_sp_(Cu(OH)_2_) = 2.20^–20^. While precipitation is a method for remediation purposes, this study focused on adsorption from solution to the magnetite surface, and hence the pH values were not higher than 5.5 for Cu^2+^.

### Kinetic Adsorption Experiments

For kinetic adsorption experiments, different treatments were prepared as above, in triplicate in the glovebox, but with a total volume of 50 mL. For each time point 2 mL of well-mixed liquid was removed, centrifuged for 2 min at 10 000*g*, and then further treated as described above to separate aqueous and solid fractions. Kinetic experiments were performed with 500 μM Cd^2+^ at pH 5.5 and 7.3 for all types of MNPs. For Cu^2+^, 750 μM was utilized at pH 5.0 with native MNPs only and at pH 5.5 with all types of MNPs. The different initial concentrations of Cd^2+^ and Cu^2+^ were selected based upon their respective adsorption isotherms that led to approximately 50% adsorption in the respective pH ranges.

### Metal Analyses

Concentrations of Cd, Cu, and Fe were determined with MP-AES, equipped with a SP3 autosampler. Samples were diluted in 2% puriss.HNO_3_ to obtain a concentration in the measurement range of the instrument. The measurement wavelengths were 371.993 nm for Fe, 228.802 nm for Cd, and 324.754 nm for Cu. The obtained data were first processed by the internal software of the instrument (MP Expert software, 1.5.0.6545).

### Specific Surface Area

Magnetite nanoparticles were anoxically freeze-dried and weighed anoxically, and then the specific surface area (SSA) was quantified with a Micromeritics Gemini VII surface area and porosity analyzer (Micromeritics Instrument Cooperation, USA), equipped with a VacPrep 061 and using N_2_ as adsorbate. SSA was only determined for mag_nat_ particles.

### Mössbauer Spectroscopy

One sample of the native magnetite was filtered in a glovebox through a 0.45 μm pore-size syringe filter (Millipore membrane), embedded in Kapton tape and stored at −20 °C until measurement. The sample was inserted into a closed-cycle exchange gas cryostat (SHI-650-5; Janis Research, USA). The spectrum was collected at 140 K using a constant acceleration drive system (WissEI, Blieskastel, Germany). γ-Radiation was emitted by a ^57^Co-source embedded in a rhodium matrix. The sample spectrum was calibrated against a 7 μm thick Fe(0) foil at room temperature. The software package recoil (University of Ottawa, Canada) was used for fitting using the extended Voigt-based fitting model. The Lorentzian half-width–half-maximum (hwhm) value was kept constant at 0.124 mm/s. The spectrum was analyzed with respect to the isomer shift (δ), the quadrupole splitting (Δ*E*_Q_), and the hyperfine magnetic field (**B**_hf_), and the Gaussian width (standard deviation) of the Δ*E*_Q_ was used to account for line broadening until the fit was reasonable.

### Micro X-ray Diffraction

Samples for micro X-ray diffraction (μ-XRD) were washed with anoxic Milli-Q and anoxically dried in an Eppendorf tube in the glovebox. μ-XRD was performed with a Bruker’s D8 Discover GADDS XRD2 microdiffractometer equipped with a standard sealed tube with a Cu-cathode (Cu Kα radiation, λ = 0.154 nm, 30 kV/30 mA). The total measurement time was 240 s at two detector positions, 15° and 40°. Phase identification was validated using Match! software version 3.7.1.123 with Crystallography Open Database (COD-Inorg REV211633 2018.19.25). μ-XRD patterns were utilized to obtain information about mineralogy and crystal size. The Scherrer equation (eq 1) was applied to calculate average crystal size *d*:^42^
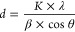
1with *K* = shape factor (0.9), λ = wavelength of the source, β = full width at half-maximum (fwhm), and cos θ the cosine of the Bragg angle θ.

### Data Treatment and Models of Isotherm and Kinetic Adsorption

The data obtained from MP-AES measurements were evaluated to obtain the amount of adsorbed contaminant as Cu/Cd in μmol on mass of Fe in g (μmol/g Fe) by calculating mean and standard deviation of technical triplicates. We used both Langmuir^43^ and Freundlich^44^ isotherms (eqs 2 and 3) for all collected data sets.
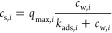
2
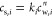
3*c*_s,*i*_ (μmol/g) represents the amount of adsorbed Cd^2+^ or Cu^2+^, *c*_w,*i*_ is the concentration in solution (μmol/L), *k*_ads,*i*_ (μmol/L) is the binding constant, and *q*_max,*i*_ (μmol/g) is the maximum adsorption capacity. *k*_*i*_ is the Freundlich adsorption coefficient [(μmol/g)(L/g)^*n*^], and *n* is the Freundlich coefficient. Here the subscript *i* always refers to the different experiments (pH/magnetite/heavy metal). Isotherms were fit using the nonlinear least-squares solver *lsqnonlin* (trust region approach)^23,45^ in MATLAB (R2022b) (objective function in *Parameter estimation*). For all the parametrizations we report the fitted parameter values and the goodness of fit of the model as normalized root-mean-square-error (NRMSE) (eq 4)^46^
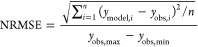
4where *n* is the number of observations and *i* the observation indices.

For kinetic experiments, the rates of adsorption of Cu^2+^ and Cd^2+^ were defined by a linear driving force^24,44^ and a second-order adsorption scheme (eqs 5 and 6).^24^ Divalent heavy metals (HM(II)) were assumed to be distributed between equilibrium *S*_HM(II)_^EQ^ and actual concentration of adsorbed Cu^2+^ or Cd^2+^ (*S*_HM(II)_) (μmol/g). This approach was previously utilized.^24,47^ Here, we applied both Langmuir and Freundlich isotherms (eqs 2 and 3) to compute the equilibrium concentration *S*_HM(II)_^EQ^. The rates of adsorption were finally formulated by multiplying the concentration differences by the empirical kinetic adsorption rates constants *k*_sorb,1_ (s^–1^) and *k*_sorb,2_ (μmol^–1^ g s^–1^) for eqs 5 and 6, respectively.
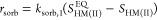
5
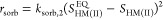
6
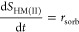
7

The ordinary differential equation (ODE) (eq 7) was solved in MATLAB using the ODE solver *ode15s*.^48^

#### Parameter Estimation

The model (eqs 2, 3, and 5–7) parameters *q*_max_, *k*_ads_, *k*, *n*, *k*_sorb,1_, and *k*_sorb,2_ were estimated. The objective function is defined in eq 8
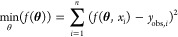
8where **θ** is the parameter vector and *y*_obs,*i*_ the observations. The *lsqnonlin* algorithm in MATLAB was used for optimization by minimizing eq 8. NRMSE was computed to evaluate the goodness of fit (eq 4).

## Results and Discussion

### Magnetite Characterization

Synthesized native magnetite mag_nat_ had a Fe(II)/Fe(III) ratio of 0.42 ± 0.01. Microbially oxidized (mag_ox_) and reduced (mag_red_) magnetite had ratios of 0.26 ± 0.02 and 0.54 ± 0.03 respectively, suggesting successful magnetite oxidation and reduction by the nitrate-reducing Fe(II)-oxidizing culture KS and Fe(III)-reducer *G. sulfurreducens*. The SSA of the freeze-dried MNPs measured with BET was 92.73 m^2^/g, which was comparable with literature.^39^ The high SSA is explained by the small size of the particles, as the described synthesis method commonly results in particles in a size order of 10 nm.^39^ Calculated apparent diameter *d*_app_(49) resulted in 12.49 nm.

^57^Fe Mössbauer analysis at 140 K confirmed that the prepared mineral was magnetite with two characteristic sextets in the spectrum correlating to Fe in octahedral and Fe in tetrahedral coordination (Figure S1 and Table S1). The Fe(II)/Fe(III) ratio was calculated according to Gorski and Scherer^27^ as 0.46 ± 0.024, which was in reasonable agreement with the ratio determined by the ferrozine assay (0.42 ± 0.01).

μ-XRD also confirmed materials used for all adsorption experiments to be magnetite (Figures S2–S4). Minor reflections corresponding to vivianite (Fe^II^_3_(PO_4_)_2_, 8H_2_O) were visible in the pattern for mag_red_ at 2Θ of 15.32°. The reduction of magnetite by *G. sulfurreducens* presumably caused partial dissolution of some Fe^2+^ which precipitated as vivianite in the PO_4_^3–^-rich medium used in this study.^50,51^ Based on the relatively low intensity of the reflections in the XRD patterns, coupled to previous measurements of the SSA of vivianite of 8–16 m^2^ /g,^52,53^ we anticipate that the effect of vivianite in this system was minor and did not influence the adsorption experiments. The Scherrer equation (eq 1) was used to calculate the average crystal size^42,54^ of 9.59 nm for mag_red_ and 10.23 nm for mag_ox_ and 10.29 nm for mag_nat_. The slight decrease of crystal size for the reduced MNPs reflected a relative change of 6.8% (0.699 nm) and of 0.53% (0.055 mm) for mag_ox_. μ-XRD patterns were collected for native MNPs after kinetic and isotherm experiments, with all results confirming pure magnetite and no vivianite (Figures S3 and S4).

Using the average crystal size obtained from the Scherrer equation, we calculated the theoretical SSA according to Etique et al.^49^ to be 107.7 m^2^ g^–1^ for mag_red_, 101.0 m^2^ g^–1^ for mag_ox_, and 100.4 m^2^ g^–1^ for mag_nat_. This suggests microbial activity influenced the SSA of the MNPs, though the differences are relatively small. Comparison to the BET-results, the measured SSA (92.73 m^2^ g^–1^) for mag_nat_ showed that the measurement and calculation are within 10% relative error. Since our calculated SSAs showed small differences overall of less than 7%, the great changes of adsorption properties cannot be explained by the changes in surface area alone.

To confirm the successful removal of biomass, the DOC content of the supernatant of the washed particles was determined. The results yielded a DOC content of 1.28 mg C/L which is just slightly above the Milli-Q water used to prepare all solutions (0.95 mg C/L). Representative fluorescence microscopy images, collected after washing oxidized and reduced MNPs 5 times (Figures S5 and S6), showed no more colored areas, suggesting successful removal of cells. Figure S7 shows the results after washing the reduced MNPs only once which shows many cells remained associated with the MNPs.

While we made every effort to wash the MNPs to be free from bacteria, we cannot guarantee that no residual organic compounds remained. The NO_3_^–^ anion is however not expected to have a significant influence on the magnetite properties because it has been shown before that the binding of metals with nitrate is minor or negligible^55^ and the adsorption of nitrate to magnetite is minor.^56,57^ We therefore propose that any influence of NO_3_^–^ on the adsorption of metal cations was systematic and not significant.

Since this study is dealing with adsorption of Cu and Cd onto nanoparticles, particle aggregation is an important process^58,59^ that could influence the available surface area and thus adsorption capacities. If any organic compounds (from biomass) remained in the magnetite solution after washing, particle aggregation could have influenced^60^ the adsorption. In a previous study^61^ the comparison of abiotically synthesized and biologically induced MNPs showed aggregation differences between biogenic MNPs (larger and less compact). However, in our study the microorganisms were not responsible for the synthesis of the MNPs, and as shown above, our MNPs were thoroughly washed and showed little evidence of any associated organic compounds, suggesting that its impact on aggregation and adsorption itself should be minor.

### Redox Potential and pH_PZC_

Gorski et al.^62^ empirically derived a linear relationship of the Fe(II)/Fe(III) ratio in magnetite and its open circuit potential (*E*_OCP_). They showed that an increase in the stoichiometry of magnetite resulted in a decrease of *E*_OCP_. Using this expression, we calculated the potential of our MNPs which resulted in −0.54, −0.36, and −0.12 mV for mag_red_, mag_nat_, and mag_ox_ respectively. This suggests that the potential in our MNPs changed over ±0.42 mV from oxidized to reduced magnetite.

Literature described the point of zero charge pH_PZC_ for magnetite at around pH 6.5.^17,63^ Therefore, we can assume that at pH 5.5, 6.5, and 7.3 mag_nat_ should have positive, almost neutral, and negatively charged surface potential at the three different pH values, respectively. We can therefore assume that the pH_PZC_ shifted relatively toward lower pH values for mag_red_ and toward higher pH values for mag_ox_.

### Adsorption Isotherms and Kinetics

#### i Copper

**Figure 1:**
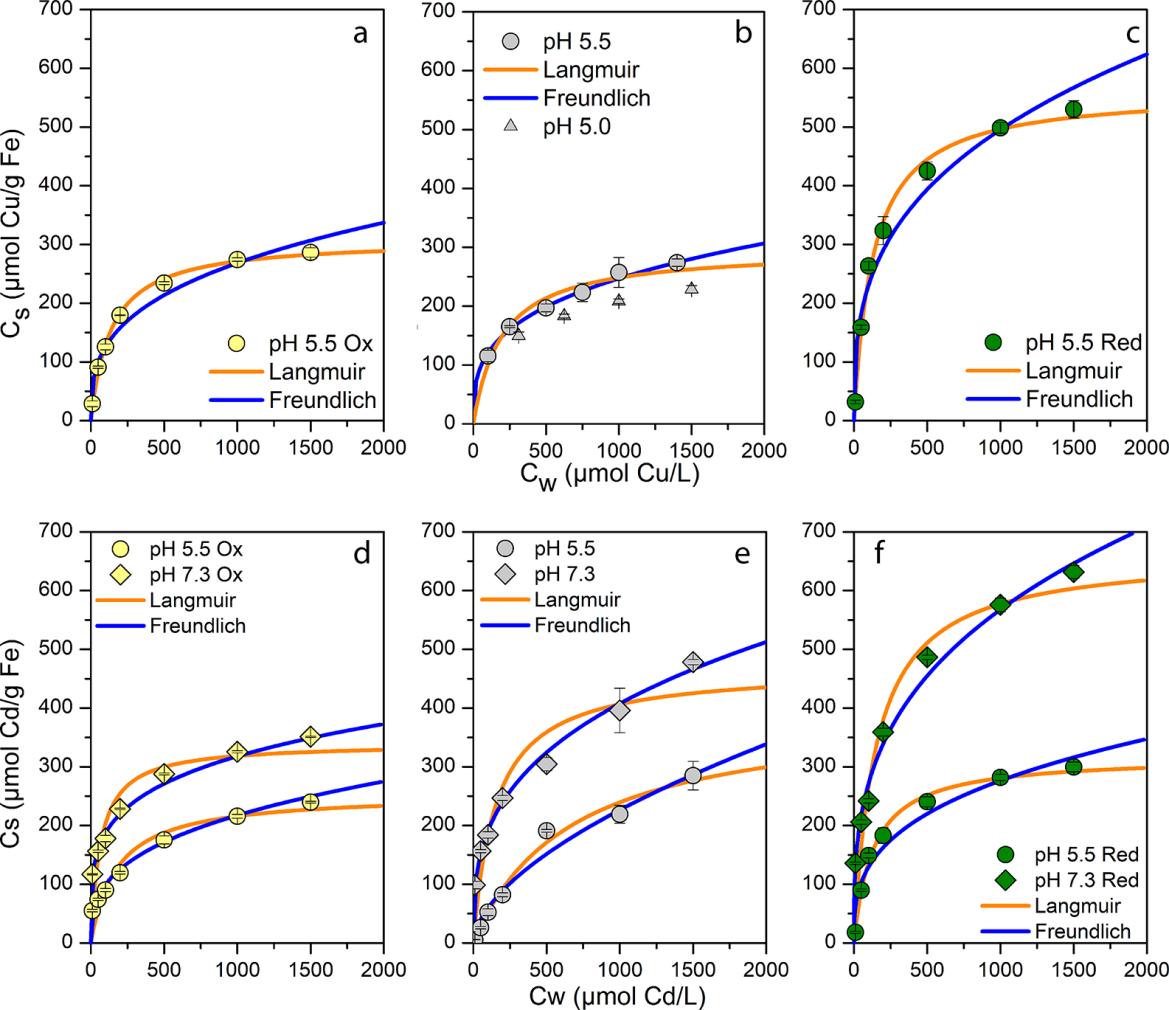
Measured data and fit isotherms for Cu^2+^ (a–c) and Cd^2+^ (d–f) adsorption at pH 5.5 (circles) with native (gray), reduced (green), and oxidized (yellow) magnetite nanoparticles. Additionally at pH 7.3 (diamonds) for Cd^2+^. Triplicate bottles with increasing Cu^2+^/Cd^2+^ concentrations were incubated for 24 h, and the amount of adsorbed Cu/Cd (in μmol) on mass of magnetite (as g Fe) was determined via MP-AES. Langmuir (orange) and Freundlich (blue) isotherms were modeled. Gray triangles for Cu^2+^ with native magnetite show results of isotherm at pH 5.0.

For mag_nat_, the maximum concentration of adsorbed Cu^2+^ increased from 228.69 ± 6.25 μmol/g Fe (pH 5.0) to 273.9 ± 6.32 μmol/g Fe (pH 5.5) (Figure 1). Adsorption experiments with oxidized and reduced magnetite were conducted with Cu^2+^ at pH 5.5. Mag_ox_ at pH 5.5 exhibited similar adsorption that was slightly increased (286.44 ± 8.01 μmol Cu/g Fe) over mag_nat_ (273.9 ± 6.32 μmol Cu/g Fe), indicating the effect of microbial oxidation of magnetite was minor. In stark contrast, mag_red_ adsorbed 530.13 ± 14.70 μmol/g Fe, which was roughly twice as much as for mag_nat_ and mag_ox_. Reduction of magnetite has been previously described to “charge” particles with electrons^28^ for both nano- and microscaled particles. This could lead to a corresponding increase in “negative charge” and decrease the point of zero charge of the magnetite and ultimately lead to a less positively charged surface. The point of zero charge (pH_PZC_) is defined as the pH where the total net charge on the surface is zero^17^ as discussed above. Below the pH_PZC_, the electrostatic repulsion effect of the same charges, here the positively charged surface of MNPs and divalent cation (Cu^2+^), decreased as the surface sites of magnetite deviated from a fully protonated surface (−FeOH_2_^+^) toward a more negatively charged surface (−FeOH^–^).^17^ The more negatively charged the surface, the more positively charged Cu^2+^ can adsorb. Alternatively, the increased adsorption capacity could be due to an increased SSA as a result of microbially induced dissolution. Without further measurements these assumptions are however only speculative, and we suggest that both mechanisms occurred.

Kinetic adsorption experiments were carried out to better understand the time dependence of Cu^2+^ adsorption to the different types of magnetite. Mag_nat_ was tested at pH 5.0 and 5.5 with little divergence in the concentration of Cu^2+^ adsorption until the final sampling time point at 24 h (Figure S8). It was expected that an increased pH would lead to increased adsorption, since the surface charge of the mineral was less negative.^17^

**Figure 2:**
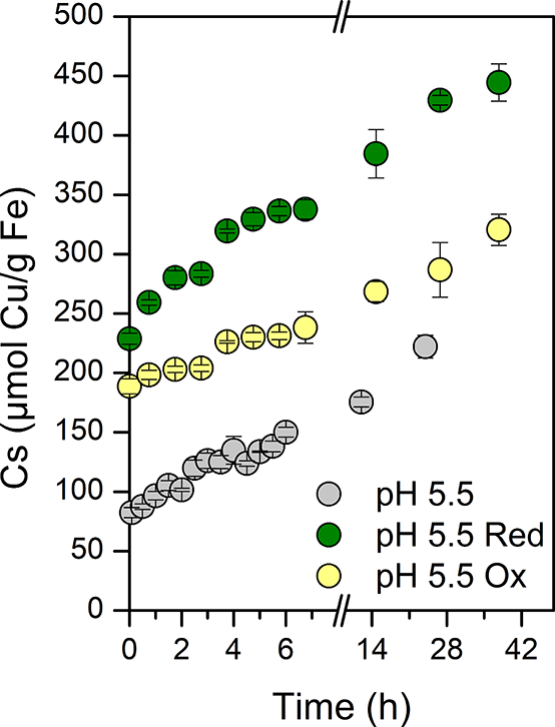
Kinetic behavior of Cu^2+^ adsorption on magnetite nanoparticles at pH 5.5 with native (gray), reduced (green), and oxidized (yellow) MNPs. Triplicate bottles were incubated with MNPs (as 9 mM Fe) and 750 μM Cu^2+^. Adsorbed Cu (μmol) on mass of magnetite (as g Fe) was regularly determined via MP-AES.

The adsorption on mag_red_ after 5 min was already 40 μmol/g Fe greater than that on mag_ox_ and 146 μmol/g Fe greater than that on mag_nat_ (Figure 2). After 1 day 429.56 ± 4.05 μmol Cu/g Fe was adsorbed on mag_red_, 286.79 ± 2.97 μmol Cu/g Fe on mag_ox_, and 222.23 ± 9.60 μmol Cu/g Fe on mag_nat_. The adsorption of Cu^2+^ on MNPs did not reach equilibrium after 24 h for intermediate and higher concentrations of dissolved Cu^2+^, as adsorption continued onto mag_red/ox_ between hours 26.75 and 37.75. The difference after a few minutes of contact time shows the importance of the stoichiometry of the MNPs (changed through microbial oxidation and reduction) on the rate of adsorption. Both mag_ox_ and mag_red_ adsorbed twice as much Cu^2+^ as mag_nat_ immediately and showed higher capacity even after 2 days.

#### ii Cadmium

Since Cd^2+^ is more soluble than Cu^2+^ across a wide pH range, Cd^2+^ adsorption isotherms to mag_nat_ were performed at pH 5.0, 5.5, 6.5, and 7.3. As expected, the amount of adsorbed Cd^2+^ on native MNPs increased with pH from 256.95 ± 45.68 μmol/g Fe (pH 5.0), 284.97 ± 24.19 μmol/g Fe (pH 5.5), 417.78 ± 16.08 μmol/g Fe (pH 6.5), to 478.20 ± 4.66 μmol/g Fe (pH 7.3) (see Figure S9). Due to the previously discussed change of positive to negative surface charge across the point of zero charge, more Cd^2+^ was able to adsorb on the native MNPs with increasing pH. Plotting the maximum of adsorbed Cd^2+^ vs pH (see Figure S10) reveals a linear relationship in the observed pH range. We assumed that further increasing pH will lead to more adsorption of Cd^2+^ onto MNPs. Based on a dissolved Cd^2+^ concentration of 1.5 mM, this shows that adsorption could be studied up to pH 8.6 without precipitation of cadmium hydroxide Cd(OH)_2_ (*K*_sp_ of Cd(OH)_2_) = 2.5^–14^). Using the linear trend shown in Figured S10, we calculated the maximum possible amount of Cd on mag_nat_ under these conditions as 610.66 μmol Cd/g Fe.

**Figure 3:**
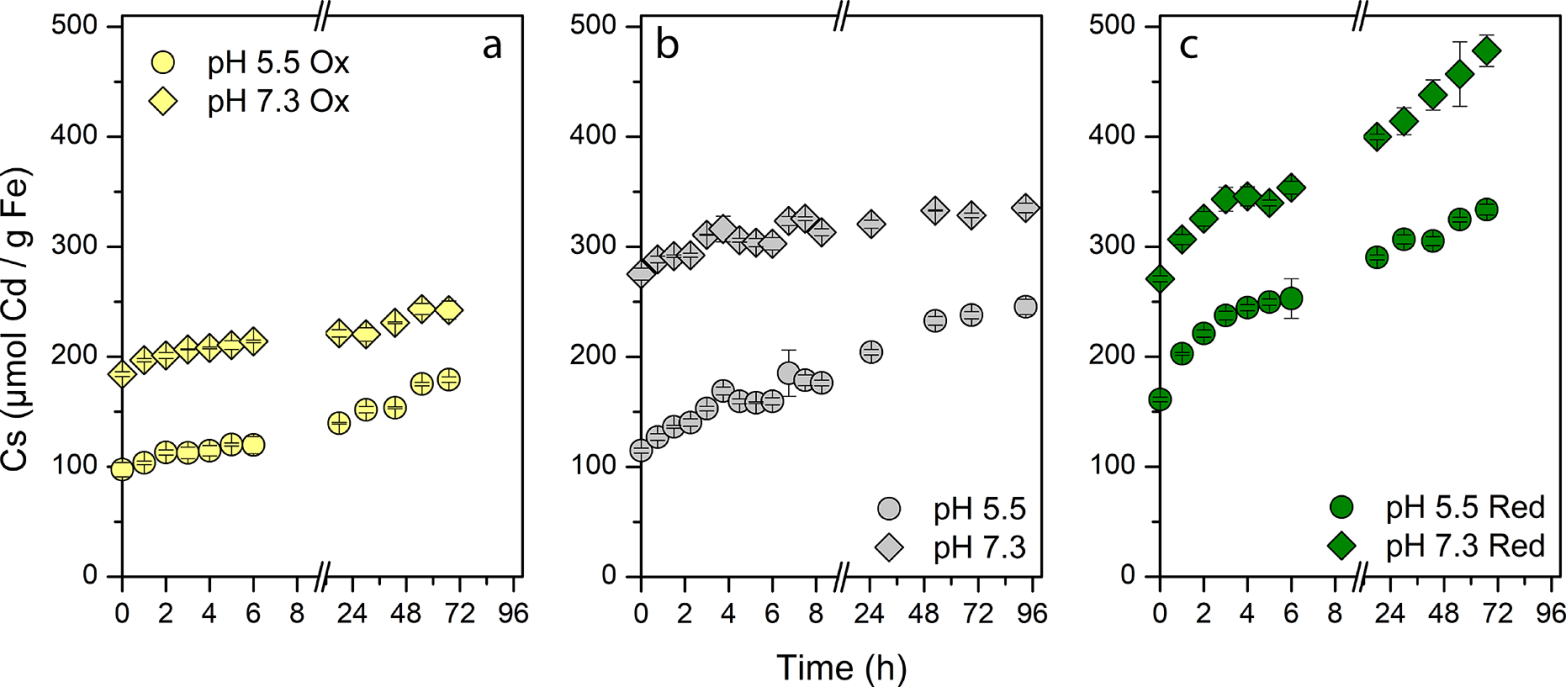
Kinetic behavior of Cd^2+^ adsorption on magnetite nanoparticles at pH 5.5 (circles) and pH 7.3 (diamonds) with native (gray, panel b), reduced (green, panel c), and oxidized (yellow, panel a) magnetite. Triplicate bottles were incubated with magnetite (as 9 mM Fe) and 500 μM Cd^2+^. Adsorbed Cd (in μmol) on mass of magnetite (as g Fe) was measured via MP-AES.

Isotherm (Figure 1) and kinetic (Figure 3) experiments were performed at pH 5.5 and 7.3 for native, oxidized, and reduced MNPs. At pH 5.5 mag_ox_ could adsorb less Cd^2+^ (239.84 ± 1.54 μmol Cd/g Fe) compared to mag_red_ (299.68 ± 8.31 μmol Cd/g Fe), which was slightly above mag_nat_ (284.97 ± 24.19 μmol Cd/g Fe) but within standard deviation of the mean. When comparing results at pH 5.5 for mag_nat_, mag_red_, and mag_ox_, the change in stoichiometry, especially when MNPs were reduced, showed a much greater effect for Cu^2+^ than Cd^2+^. Even though both Cu^2+^ and Cd^2+^ are divalent cations, Cd^2+^ has a much bigger radius of 109 pm, while the Cu^2+^ radius is only 87 pm. Steric interactions and repulsion of larger Cd^2+^—Cd^2+^ ions in solution, paired with a still positively charged surface of MNPs even after reduction at low pH, could explain this difference.^64−66^

**Figure 4:**
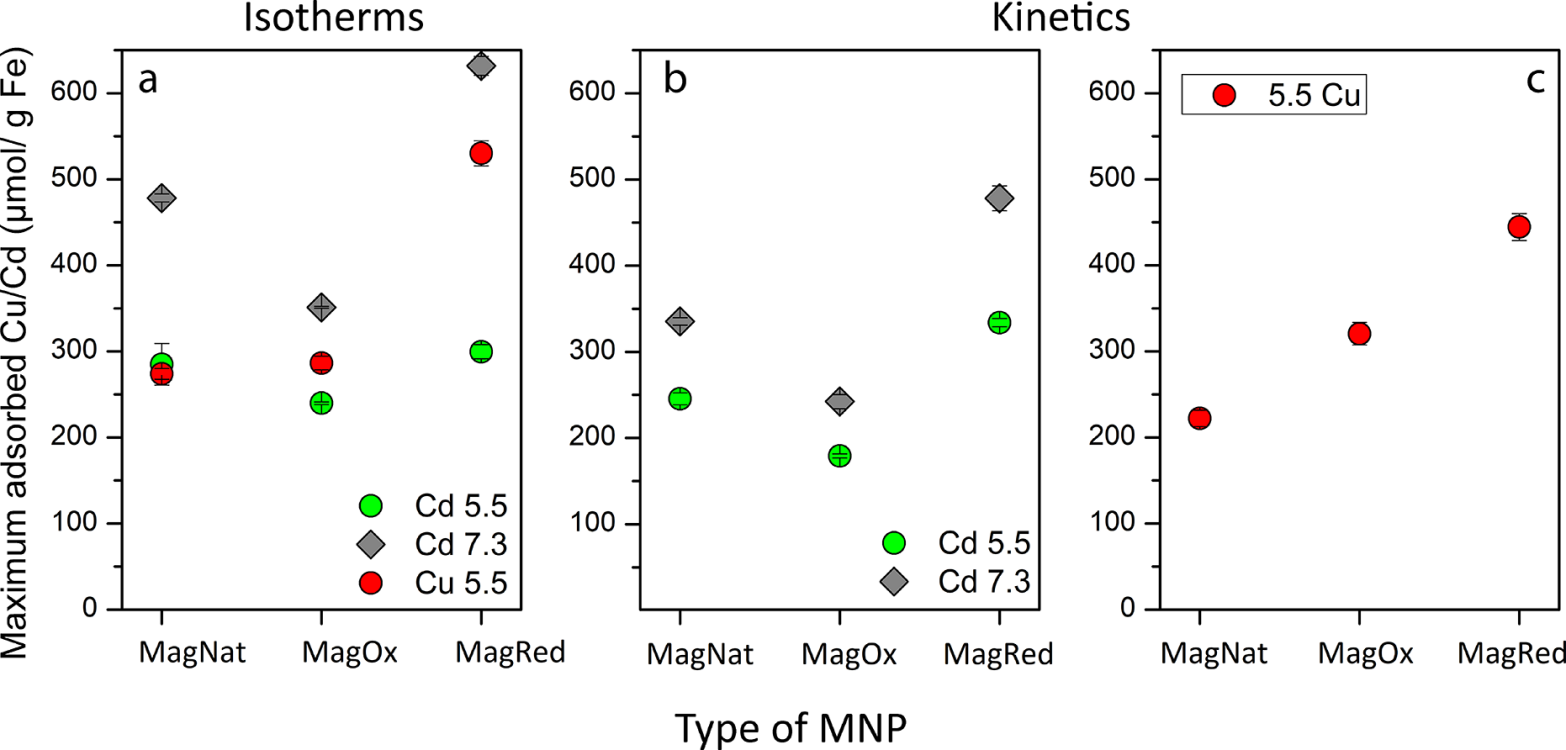
Summary of maximum adsorbed heavy metal concentrations for isotherm experiments (a) and kinetic experiments (b, c) with MNPs. MNPs were untreated (native: MagNat) or microbially oxidized (MagOx) or reduced (MagOx). Displayed are the results as μmol heavy metal/g Fe ± standard deviation for Cd^2+^ at pH 5.5 (light green circles) and pH 7.3 (dark gray diamonds) and Cu^2+^ at pH 5.5 (red circles).

At pH 7.3, mag_ox_ showed the lowest removal capacity toward Cd^2+^ with 351.19 ± 1.14 μmol Cd/g Fe, followed by mag_nat_ with 478.20 ± 4.66 μmol Cd/g Fe, and surpassed by mag_red_ with 631.72 ± 11.00 μmol Cd/g Fe. The increased pH led to a less positively charged surface area, and hence more divalent cations could adsorb. Interestingly, at pH 7.3 the stoichiometry of MNPs had a greater influence than at pH 5.5 as seen by the greater adsorption by mag_red_, which was also reflected in the difference between maximum adsorption of Cd at pH 5.5 and pH 7.3 (Figure 4). The increase of adsorbed Cd^2+^ from pH 5.5 to 7.3 was 111.36 μmol Cd/g Fe for mag_ox_, 193.23 μmol Cd/g Fe for mag_nat_, and 332.04 μmol Cd/g Fe for mag_red_. At higher pH both the Fe(II)-enriched negatively charged magnetite surface area and the more negatively charged bulk mineral yielded higher Cd^2+^ adsorption. Independently of pH the oxidation of MNPs showed a decrease in adsorption capacity toward Cd^2+^. We suggest that the increase in positive charge of the MNPs exhibits a repulsive force on the Cd^2+^ ions. The change in stoichiometry of the MNPs played an important role at high pH values for Cd^2+^, while the effect of pH dominated at pH 5.5 and an influence of the stoichiometry could still be detected at pH 5.5 that resulted in increased adsorption for mag_red_ and decreased adsorption for mag_ox_.

Results from the kinetic experiments for Cd^2+^ ([Cd^2+^] = 500 μM) performed with all MNPs at pH 5.5 and 7.3 confirmed the previously discussed findings and expanded on them (Figure 3). At both pH values, the adsorption of Cd^2+^ on mag_ox_ showed the slowest rate and achieved the lowest total amount after more than 2 days. Rate and amount of adsorbed Cd^2+^ on reduced MNPs were greater when compared to native MNPs. Interestingly, the amount of adsorbed Cd^2+^ at this intermediate concentration in solution (initially 500 μM Cd^2+^) on mag_red_ at pH 5.5 reached roughly the same value (333.84 ± 4.85 μmol Cd/g Fe) after only 67 h as the adsorbed Cd^2+^ on mag_nat_ at pH 7.3 after 96 h (335.35 ± 4.23 μmol Cd/g Fe). This showed that for Cd^2+^ adsorption on MNP, the reduction led to an increase of the adsorption rate and capacity. Our results showed the same trends for pH 7.3. Mag_ox_ adsorption was smallest, followed by mag_nat_, and then surpassed by mag_red_. We can see in Figure 3 that the amount of adsorbed Cd^2+^ on mag_nat_ after 12 h did not increase much further, while the amount on mag_red_ continued to increase until the last sampling time point. This suggests that independent of the pH the oxidation of MNPs greatly hinders the adsorption of Cd^2+^ while reduction greatly increased it. At low pH there was little difference in the performance of mag_nat_ or mag_ox_ with respect to Cd^2+^. However, almost immediately 161.15 μmol Cd/g Fe was adsorbed by mag_red_ (Figure 3), which was 1.4× as much as mag_nat_ with 114.88 μmol Cd/g Fe and 1.7× as much as mag_ox_ with 97.34 μmol Cd/g Fe at the same time point. Therefore, reduced MNPs provide enhanced adsorption even for short contact times and low pH values. Additionally, mag_red_ initially adsorbed 270.86 μmol/g Fe at pH 7.3, which was more than mag_ox_ (184.14 μmol Cd/g Fe) but similar to mag_nat_ (275.16 μmol Cd/g Fe). Adsorption to mag_ox_ remained low by the end of the study (242.41 μmol Cd/g Fe) whereas adsorption on mag_nat_ increased to 335.35 μmol/g Fe and to 478.15 μmol/g Fe for mag_red_.

Figure 4 summarizes the maximum measured adsorbed amount of Cd^2+^/Cu^2+^ on the different redox MNPs. We show the maximum adsorbed amount for isotherms (left panel, same *c*_w_ concentration range for Cu^2+^ and Cd^2+^) and the kinetic experiments (right two panels, different *c*_w_ for Cd^2+^ and Cu^2+^ during kinetic experiments) but only discuss the numbers of the isotherms and use the kinetic data to support these findings. We can see that for mag_nat_ at pH 5.5 the amount of adsorbed Cu^2+^ and Cd^2+^ was within standard deviation, suggesting that at this pH all available surface sites of the unaltered magnetite were saturated for both heavy metals. At pH 7.3 (only Cd^2+^) about 159 additional μmol Cd/g Fe was adsorbed for mag_nat_, showing the importance of pH for adsorption processes (see also Figure S9). For mag_ox_, the adsorption of Cu^2+^ increased slightly compared to mag_nat_. More Cu^2+^ than Cd^2+^ was adsorbed on mag_ox_, since the amount of adsorbed Cd^2+^ slightly decreased from mag_nat_ to mag_ox_ which was in contrast with the slight increase for Cu^2+^. This suggests that previously occupied surfaces sites were not available anymore for Cd^2+^ but remained available for Cu^2+^. Additionally, a more positively “redox-discharged” mineral (decreased Fe(II)/Fe(III) ratio) likely increased electrostatic repulsion toward the bigger cation Cd^2+^ more profoundly than for smaller Cu^2+^. Previously the oxidation of magnetite was reported as a surface sensitive process,^28,67^ and hence this positively charged surface would repel Cd^2+^. This is reflected in the low adsorption of Cd^2+^ with mag_ox_ at pH 7.3. These findings were supported by kinetic experiments, which consistently showed smaller *c*_s_ values for Cd^2+^ on mag_ox_ than for mag_nat_ and greater *c*_s_ values for Cu^2+^ with mag_ox_ than for mag_nat_. For Cd^2+^ at pH 7.3, the amount of adsorbed Cd^2+^ on mag_ox_ decreased by roughly 127 μmol Cd/g Fe compared to mag_nat_ and conclusively only increased by roughly 66 μmol Cd/g Fe compared to pH 5.5 mag_ox_, showing the importance of the minerals’ stoichiometry. Considering Figure S10, the theoretical maximum *c*_s_ of Cd^2+^ was calculated as 610.60 μmol Cd/g Fe at pH 8.6, just before precipitation of Cd(OH)_2_. At pH 7.3, mag_red_ already showed a higher *c*_s_ of 631.72 ± 11.00 μmol Cd/g Fe, again emphasizing the great importance of MNPs’ stoichiometry. The difference at pH 7.3 between mag_nat_ and mag_ox_ was more profound than for pH 5.5, as more Cd^2+^ was adsorbed to the minerals’ surface at pH 7.3 to begin with. Additionally, it appears that the impact of surface charge is more important at low pH than the stoichiometry for Cd^2+^ and that the stoichiometry gains importance as pH rises. This was also supported by the kinetic experiments (Figure 3) where we consistently measured increasing *c*_s_ values of Cd^2+^ in the order of mag_ox_ < mag_nat_ < mag_red_. For mag_red_ the amount of adsorbed Cd^2+^ at pH 5.5 was within error of mag_ox_ and slightly greater than mag_nat_, supporting the hypothesis that the adsorption process in the system was mostly influenced by pH. A low pH value led to a positive surface charge of the MNPs, as the pH_PZC_ was previously reported between 6.1 and 8 for magnetite.^17,67^ Interestingly adsorption of Cu^2+^ on mag_red_ was almost doubled to 530 μmol Cu/g Fe (see also Figure 1) compared to mag_nat_ at pH 5.5. It appears that the net negative charge of the “bulk” magnetite^28^ influenced the adsorption of the smaller cation Cu^2+^ at lower pH more intensely than for the bigger Cd^2+^ cation already at pH 5.5. With mag_red_ the amount of adsorbed Cd^2+^ only increased within standard deviation at pH 5.5 while the amount of Cd^2+^ adsorbed on mag_red_ at pH 7.3 increased to 631.72 ± 11.00 μmol Cd/g Fe, which was 153 μmol Cd/g Fe greater than on mag_nat_. While the pH had greater influence on the adsorption of Cd^2+^ onto the MNPs surface at low pH values for Cd^2+^, increase to pH 7.3 revealed the importance of MNPs’ stoichiometry as the adsorption capacity was decreased for mag_ox_ and increased for mag_red_, both compared to mag_nat_, which was again consistent with the kinetic data (Figure 3, Figure 4 right panels). It was previously reported^33^ that the stoichiometry of magnetite is a key parameter for the binding of emerging organic contaminants and naturally ligands, as they showed for nalixidic acid the importance of redox for removal of Cr and As.^22^ We add on to this knowledge by showing that the stoichiometry of magnetite is crucial for the removal of different divalent heavy metals and that it can have a greater impact than change of pH.

#### Importance of Contact Time

While the isotherm experiments with Cu^2+^ (Figure 1) indicated that the time frame of 24 h was sufficient, the kinetic experiments revealed that the adsorbed amount of Cu^2+^ still increased, especially for mag_ox_ and mag_red_, even after 42 h (Figure 2). Therefore, a longer contact time would be needed in order to obtain equilibrium. The isotherms collected for Cd^2+^ showed that, especially at high pH values and with mag_nat_ and mag_red_, the contact time of 24 h was insufficient (Figure 1 and Figure 3). As we could show with the kinetics experiment for Cd^2+^ at pH 5.5 and 7.3 for all MNPs, a contact time of 24 h was sufficient for mag_nat_, but at least 48 h was needed for mag_red_ and mag_ox_. We therefore recommend a contact time greater than 48 h to explore the future potential of microbially enhanced MNPs for heavy metal removal.

### Modeling

#### Kinetic Experiments

The results and corresponding parameters of the kinetic experiments are shown in Figures S11–S12 and Tables S3–S5. Since the experiments with Cu^2+^ were performed in a narrow range of pH, all kinetic experiments could be modeled using a first- or second-order rate with a NRMSE < 0.06. Data presented in Figure S11 and Table S3–S5 suggested that the collected data could be fitted well with both Langmuir or Freundlich equilibria and first- or second-order kinetics. However, a second-order scheme seemed slightly more suitable for Cu^2+^ with all types of MNPs at both investigated pH values for both equilibrium isotherms (Langmuir or Freundlich). This could indicate that the adsorption of Cu^2+^ onto MNPs is governed by a chemisorption process, which would then have been the rate-determining step.^68^ Previous studies on adsorption of Cu^2+^ onto magnetite have reported that second-order kinetics was a superior model.^35^ For Cd^2+^, no good modeled results were obtained for mag_nat_ at pH 5.5, suggesting that the collected data were of inferior quality compared to the other data set, which could also be implied by (comparatively) large standard deviation of the mean. Additionally, mag_nat_ at pH 7.3 with Cd^2+^ also did not yield a good modeled results; while the model parameters could be bent to fit the data (Figure S12), the parameter results presented in Tables S4 and S5 were not reasonable. In summary, there was not a clear trend in favor of one specific model, and hence either Langmuir or Freundlich as first- or second-order kinetics could be used.

#### Adsorption Isotherms

The results of the modeled isotherms can be seen in Table S2 and in Figure 1. The results suggest that for Cu^2+^ a Freundlich model was a better fit for the pH 5.5 isotherms with mag_nat_ while mag_ox_ and mag_red_ were better estimated by a Langmuir equilibrium. Enhanced adsorption due to oxidation and reduction enabled higher *c*_s_ (adsorbed amount) values which then allowed better estimation of *q*_max_. Freundlich isotherms seemed to overestimate concentrations of Cu^2+^, if *c*_w_ (concentration in solution) would be increased further. For Cd^2+^ with mag_nat_, a Langmuir model fit better but for pH 7.3 a Freundlich isotherm was more appropriate (as seen by NRMSE). For Cd^2+^ at pH 5.5 and 7.3 with all types of MNPs, both Langmuir and Freundlich fits were suitable (Figure 1). Most models had a NRMSE of <0.1. Cd^2+^ isotherms generally followed a Freundlich model, which showed consistency in increasing *k* (distribution coefficient) for increasing pH of native magnetite (pH 5.0, 5.5, 6.5, 7.3: 2.52, 4.12, 22.20, 41.72, respectively) and for increasing pH for reduced and oxidized magnetite (pH 5.5 and 7.3, mag_red_: 28.28, 62.24, mag_ox_: 20.75, 64.90). Here the model however does not result in appropriate *k* values, where mag_red_ showed much higher total adsorption than mag_ox_. This was better modeled following the Langmuir equation, and we obtained appropriate *q*_max_ values for Cd at pH 7.3: mag_red_: 663.7 μmol/g Fe and mag_ox_: 339.7 μmol/g Fe.

Overall, both heavy metals could be characterized by either Langmuir or Freundlich isotherms at equilibrium. Table S2 shows the NRMSE of all experiments. The goodness of fits at different isotherm varied marginally. For the kinetics, both first- and second-order rates were tested with both Langmuir and Freundlich equilibrium assumptions, and all combinations could reproduce the dynamics in the data well (NRMSE in Table S3). Finally, while it depended on the investigated experiment which model fit best, we could parametrize a reasonable model that fits (almost) all data sets.

## Conclusions

We investigated native, microbially oxidized and microbially reduced magnetite nanoparticles (MNPs) for the amount and rate of adsorption toward the two divalent heavy metals Cd^2+^ and Cu^2+^. Our results presented here show that the influence of microbial oxidation and reduction of Fe in these MNPs greatly influences the adsorption behavior of these environmentally relevant metals. For Cu^2+^ we showed that the reduction of MNPs leads to an increase in adsorption capacity. This was expected since the reduction likely led to an increased negative bulk charge of the MNPs as we could show with potential calculations (Table S6). Additionally partial dissolution, as shown by μXRD, led to an increase in SSA of the particles (Table S6). Even the oxidized MNPs showed an increase in adsorption toward dissolved Cu^2+^ with respect to native MNPs, a phenomenon that we are unable to fully explain even when considering the slight differences in calculated SSA. As the redox potential of oxidized MNPs is higher, repulsion due to the same charges was expected to be a dominating factor during adsorption. It was assumed that the change in stoichiometry toward Fe(III) (i.e., more positively charged MNPs) would lead to a decrease in adsorption capacity and efficiency through charge repulsion. Our isotherm and kinetic experiments however showed that the opposite is true. Possibly vacancies in the mineral due to reorganization within the crystal structure^26^ could have given smaller Cu^2+^ ions (87 pm ionic radius) more available adsorption sites. On the other hand, we showed that the increase in Fe(II)/Fe(III) ratio in magnetite due to magnetite reduction resulted in almost 2 times greater adsorption of 663.7 μmol/g Fe than for mag_nat_. For Cd^2+^, we could see that at low pH values, the stoichiometry of the MNPs had a minor effect on the adsorption behavior, most likely because the greater ionic radius of Cd^2+^ (109 pm) was repelled due to the same charge from the positively charged magnetite surface, even if the “bulk” was more negatively charged after reduction. This could explain the minor increase of adsorption of MNPs at pH 5.5 for mag_red_ and the detectable decrease for mag_ox_. At higher pH, we showed that the oxidation of MNPs led to a more pronounced decreased adsorption capacity and rate even compared to native MNPs. Furthermore, reduction of MNPs led to an increase of adsorbed Cd on mag_red_ compared to mag_nat_ and mag_ox_. Our results show that ultimately both pH and stoichiometry are highly important parameters for the adsorption processes on MNPs. For relatively small divalent cations like Cu^2+^, stoichiometry had an impact at low pH values, and both microbial oxidation and microbial reduction enhanced the adsorption capacity. For larger ions like Cd^2+^, electrostatic repulsion seemed to be the dominant process at low pH, where stoichiometry mattered less, but oxidation and reduction had great influences at higher pH values.

The MNPs used in this study were cleaned from biomass prior to experiments; however, in nature such “clean” MNPs are not expected to exist. Instead, MNPs are more likely associated with biomass from bacteria (e.g., Fe(II)-oxidizing or Fe(III)-reducing bacteria) or other redox active compounds such as natural organic matter. This associated biomass could potentially have a great influence on the adsorption of Cu^2+^ and Cd^2+^ by, among other effects, blocking surface sites,^69^ changing surface charge,^70^ or influencing the particle aggregation.^61^ Therefore, to better understand the importance of biologically reduced and oxidized MNPs in the environment, further comparative studies should be performed to investigate the role of this naturally occurring biomass and its impact on the ability of bioreduced and bio-oxidized MNPs to adsorb Cu^2+^, Cd^2+^, or other metals.

Finally, our results show that the biomodification of magnetite nanoparticles could be of great use for remediation purposes and drinking water purification. However, it seems that not one material can be applied for all contaminations and all conditions, but that the environment of adsorption (microbial oxidation or reduction) and the pH of the systems must be evaluated and chosen depending on which heavy metal should be remediated most efficiently.

## Supporting Information Available

Mössbauer spectrum and fitting results, additional μ-XRD patterns of kinetic experiments, fluorescence microscopy images, kinetic and isotherm figures, linear pH–adsorption fit, Matlab data and model of kinetic experiments and their fitting parameters, and a summary of properties of MNPs (PDF)
Cadmium and copper redox magnetite isotherms, cadmium and copper kinetics, μXRD, and Mössbauer data (XLSX)

## References

[ref1] Ali H.; Khan E.; Ilahi I. Environmental Chemistry and Ecotoxicology of Hazardous Heavy Metals: Environmental Persistence, Toxicity, and Bioaccumulation. J. Chem. 2019, 2019, 6730305 10.1155/2019/6730305.

[ref2] WHO. Guidelines for drinking-water quality, 4th ed., first addendum; World Health Organization: Genenva, Switzerland, 2017. 28759192

[ref3] Imseng M.; Wiggenhauser M.; Keller A.; Müller M.; Rehkämper M.; Murphy K.; Kreissig K.; Frossard E.; Wilcke W.; Bigalke M. Fate of Cd in agricultural soils: a stable isotope approach to anthropogenic impact, soil formation, and soil-plant cycling. Environ. Sci. Technol. 2018, 52 (4), 1919–1928. 10.1021/acs.est.7b05439. 29308892

[ref4] Rozada F.; Otero M.; Morán A.; García A. Adsorption of heavy metals onto sewage sludge-derived materials. Bioresour. Technol. 2008, 99 (14), 6332–6338. 10.1016/j.biortech.2007.12.015. 18234495

[ref5] Young S. D. Chemistry of heavy metals and metalloids in soils. In Heavy metals in soils; Springer: New York, 2013; pp 51–95.

[ref6] Grant C. A.; Sheppard S. C. Fertilizer Impacts on Cadmium Availability in Agricultural Soils and Crops. Human and Ecological Risk Assessment: An International Journal 2008, 14 (2), 210–228. 10.1080/10807030801934895.

[ref7] Hutton M. Sources of cadmium in the environment. Ecotoxicology and environmental safety 1983, 7 (1), 9–24. 10.1016/0147-6513(83)90044-1. 6303746

[ref8] Nolan K. R. Copper toxicity syndrome. J. Orthomolecular Psychiatry 1983, 12 (4), 270–282.

[ref9] Gaetke L. M.; Chow C. K. Copper toxicity, oxidative stress, and antioxidant nutrients. Toxicology 2003, 189 (1–2), 147–163. 10.1016/S0300-483X(03)00159-8. 12821289

[ref10] Brun L.; Maillet J.; Hinsinger P.; Pepin M. Evaluation of copper availability to plants in copper-contaminated vineyard soils. Environmental pollution 2001, 111 (2), 293–302. 10.1016/S0269-7491(00)00067-1. 11202733

[ref11] Shannon M. A.; Bohn P. W.; Elimelech M.; Georgiadis J. G.; Mariñas B. J.; Mayes A. M. Science and technology for water purification in the coming decades. Nature 2008, 452 (7185), 301–310. 10.1038/nature06599. 18354474

[ref12] Crini G. Non-conventional low-cost adsorbents for dye removal: a review. Bioresource technology 2006, 97 (9), 1061–1085. 10.1016/j.biortech.2005.05.001. 15993052

[ref13] Fu F.; Wang Q. Removal of heavy metal ions from wastewaters: a review. Journal of environmental management 2011, 92 (3), 407–418. 10.1016/j.jenvman.2010.11.011. 21138785

[ref14] Driehaus W.; Jekel M.; Hildebrandt U. Granular ferric hydroxide—a new adsorbent for the removal of arsenic from natural water. J. Water Supply: Res. Technol. Aqua 1998, 47 (1), 30–35. 10.2166/aqua.1998.0005.

[ref15] Berg M.; Luzi S.; Trang P. T. K.; Viet P. H.; Giger W.; Stüben D. Arsenic removal from groundwater by household sand filters: comparative field study, model calculations, and health benefits. Environ. Sci. Technol. 2006, 40 (17), 5567–5573. 10.1021/es060144z. 16999141

[ref16] Van Le A.; Straub D.; Planer-Friedrich B.; Hug S. J.; Kleindienst S.; Kappler A. Microbial communities contribute to the elimination of As, Fe, Mn, and NH4+ from groundwater in household sand filters. Science of The Total Environment 2022, 838, 156496 10.1016/j.scitotenv.2022.156496. 35667433

[ref17] Cornell R. M.; Schwertmann U. The iron oxides: Structure, properties, reactions, occurrences and uses; John Wiley & Sons: New York, 2003.

[ref18] Evans M.; Heller F. Environmental magnetism: Principles and applications of enviromagnetics; Elsevier: Amsterdam, 2003.

[ref19] Lovley D. R.; Stolz J. F.; Nord G. L. Jr; Phillips E. J. Anaerobic production of magnetite by a dissimilatory iron-reducing microorganism. Nature 1987, 330 (6145), 252 10.1038/330252a0.

[ref20] Dippon U.; Pantke C.; Porsch K.; Larese-Casanova P.; Kappler A. Potential function of added minerals as nucleation sites and effect of humic substances on mineral formation by the nitrate-reducing Fe (II)-oxidizer Acidovorax sp. BoFeN1. Environ. Sci. Technol. 2012, 46 (12), 6556–6565. 10.1021/es2046266. 22642801

[ref21] Jiao Y.; Kappler A.; Croal L. R.; Newman D. K. Isolation and characterization of a genetically tractable photoautotrophic Fe(II)-oxidizing bacterium, Rhodopseudomonas palustris strain TIE-1. Appl. Environ. Microbiol. 2005, 71 (8), 4487–4496. 10.1128/AEM.71.8.4487-4496.2005. 16085840 PMC1183355

[ref22] Sundman A.; Vitzthum A.-L.; Adaktylos-Surber K.; Figueroa A. I.; van der Laan G.; Daus B.; Kappler A.; Byrne J. M. Effect of Fe-metabolizing bacteria and humic substances on magnetite nanoparticle reactivity towards arsenic and chromium. J. Hazard. Mater. 2020, 384, 121450 10.1016/j.jhazmat.2019.121450. 31759758

[ref23] Sorwat J.; Mellage A.; Kappler A.; Byrne J. M. Immobilizing magnetite onto quartz sand for chromium remediation. J. Hazard. Mater. 2020, 400, 123139 10.1016/j.jhazmat.2020.123139. 32563903

[ref24] Sorwat J.; Mellage A.; Maisch M.; Kappler A.; Cirpka O. A.; Byrne J. M. Chromium (VI) removal kinetics by magnetite-coated sand: small-scale flow-through column experiments. J. Hazard. Mater. 2021, 415, 125648 10.1016/j.jhazmat.2021.125648. 34088175

[ref25] Byrne J. M.; Klueglein N.; Pearce C.; Rosso K. M.; Appel E.; Kappler A. Redox cycling of Fe(II) and Fe(III) in magnetite by Fe-metabolizing bacteria. Science 2015, 347 (6229), 1473–1476. 10.1126/science.aaa4834. 25814583

[ref26] Usman M.; Byrne J.; Chaudhary A.; Orsetti S.; Hanna K.; Ruby C.; Kappler A.; Haderlein S. Magnetite and green rust: synthesis, properties, and environmental applications of mixed-valent iron minerals. Chem. Rev. 2018, 118 (7), 3251–3304. 10.1021/acs.chemrev.7b00224. 29465223

[ref27] Gorski C. A.; Scherer M. M. Determination of nanoparticulate magnetite stoichiometry by Mossbauer spectroscopy, acidic dissolution, and powder X-ray diffraction: A critical review. Am. Mineral. 2010, 95 (7), 1017–1026. 10.2138/am.2010.3435.

[ref28] Byrne J. M.; van der Laan G.; Figueroa A. I.; Qafoku O.; Wang C.; Pearce C. I.; Jackson M.; Feinberg J.; Rosso K. M.; Kappler A. Size dependent microbial oxidation and reduction of magnetite nano- and micro-particles. Sci. Rep 2016, 6, 30969 10.1038/srep30969. 27492680 PMC4974511

[ref29] He Y. T.; Traina S. J. Cr(VI) reduction and immobilization by magnetite under alkaline pH conditions: the role of passivation. Environ. Sci. Technol. 2005, 39 (12), 4499–4504. 10.1021/es0483692. 16047786

[ref30] Crean D. E.; Coker V. S.; van der Laan G.; Lloyd J. R. Engineering Biogenic Magnetite for Sustained Cr(VI) Remediation in Flow-through Systems. Environ. Sci. Technol. 2012, 46 (6), 3352–3359. 10.1021/es2037146. 22397548

[ref31] Peterson M. L.; White A. F.; Brown G. E.; Parks G. A. Surface passivation of magnetite by reaction with aqueous Cr (VI): XAFS and TEM results. Environ. Sci. Technol. 1997, 31 (5), 1573–1576. 10.1021/es960868i.

[ref32] Klausen J.; Troeber S. P.; Haderlein S. B.; Schwarzenbach R. P. Reduction of substituted nitrobenzenes by Fe (II) in aqueous mineral suspensions. Environ. Sci. Technol. 1995, 29 (9), 2396–2404. 10.1021/es00009a036. 22280284

[ref33] Cheng W.; Marsac R.; Hanna K. Influence of magnetite stoichiometry on the binding of emerging organic contaminants. Environ. Sci. Technol. 2018, 52 (2), 467–473. 10.1021/acs.est.7b04849. 29215874

[ref34] Gorski C. A.; Scherer M. M. Influence of magnetite stoichiometry on FeII uptake and nitrobenzene reduction. Environ. Sci. Technol. 2009, 43 (10), 3675–3680. 10.1021/es803613a. 19544872

[ref35] Zhang J.; Lin S.; Han M.; Su Q.; Xia L.; Hui Z. Adsorption properties of magnetic magnetite nanoparticle for coexistent Cr (VI) and Cu (II) in mixed solution. Water 2020, 12 (2), 446 10.3390/w12020446.

[ref36] Kim Y.; Lee B.; Yi J. Preparation of functionalized mesostructured silica containing magnetite (MSM) for the removal of copper ions in aqueous solutions and its magnetic separation. Sep. Sci. Technol. 2003, 38 (11), 2533–2548. 10.1081/SS-120022286.

[ref37] Straub K. L.; Benz M.; Schink B.; Widdel F. Anaerobic, nitrate-dependent microbial oxidation of ferrous iron. Appl. Environ. Microbiol. 1996, 62 (4), 1458–1460. 10.1128/aem.62.4.1458-1460.1996. 16535298 PMC1388836

[ref38] Nordhoff M.; Tominski C.; Halama M.; Byrne J. M.; Obst M.; Kleindienst S.; Behrens S.; Kappler A. Insights into nitrate-reducing Fe(II) oxidation mechanisms through analysis of cell-mineral associations, cell encrustation, and mineralogy in the chemolithoautotrophic enrichment culture KS. Appl. Environ. Microbiol. 2017, 83 (13), e00752–00717. 10.1128/AEM.00752-17. 28455336 PMC5478975

[ref39] Pearce C. I.; Qafoku O.; Liu J.; Arenholz E.; Heald S. M.; Kukkadapu R. K.; Gorski C. A.; Henderson C. M. B.; Rosso K. M. Synthesis and properties of titanomagnetite (Fe 3– xTixO 4) nanoparticles: A tunable solid-state Fe (II/III) redox system. J. Colloid Interface Sci. 2012, 387 (1), 24–38. 10.1016/j.jcis.2012.06.092. 22939255

[ref40] Tominski C.; Heyer H.; Lösekann-Behrens T.; Behrens S.; Kappler A. Growth and population dynamics of the anaerobic Fe(II)-oxidizing and nitrate-reducing enrichment culture KS. Appl. Environ. Microbiol. 2018, 84 (9), e02173–02117. 10.1128/AEM.02173-17. 29500257 PMC5930324

[ref41] Stookey L. L. Ferrozine - a new spectrophotometric reagent for iron. Anal. Chem. 1970, 42 (7), 779–781. 10.1021/ac60289a016.

[ref42] Patterson A. L. The Scherrer Formula for X-Ray Particle Size Determination. Phys. Rev. 1939, 56 (10), 978–982. 10.1103/PhysRev.56.978.

[ref43] Langmuir I. The constitution and fundamental properties of solids and liquids. Part I. Solids. Journal of the American chemical society 1916, 38 (11), 2221–2295. 10.1021/ja02268a002.

[ref44] El Bardiji N.; Ziat K.; Naji A.; Saidi M. Fractal-like kinetics of adsorption applied to the solid/solution interface. ACS omega 2020, 5 (10), 5105–5115. 10.1021/acsomega.9b04088. 32201797 PMC7081449

[ref45] Coleman T. F.; Li Y. An interior trust region approach for nonlinear minimization subject to bounds. SIAM Journal on optimization 1996, 6 (2), 418–445. 10.1137/0806023.

[ref46] Liu H.; Maghoul P.; Shalaby A.; Bahari A.; Moradi F. Integrated approach for the MASW dispersion analysis using the spectral element technique and trust region reflective method. Computers and Geotechnics 2020, 125, 103689 10.1016/j.compgeo.2020.103689.

[ref47] Mikutta C.; Wiederhold J. G.; Cirpka O. A.; Hofstetter T. B.; Bourdon B.; Von Gunten U. Iron isotope fractionation and atom exchange during sorption of ferrous iron to mineral surfaces. Geochim. Cosmochim. Acta 2009, 73 (7), 1795–1812. 10.1016/j.gca.2009.01.014.

[ref48] Shampine L. F.; Reichelt M. W. The matlab ode suite. SIAM journal on scientific computing 1997, 18 (1), 1–22. 10.1137/S1064827594276424.

[ref49] Etique M.; Jorand F. P.; Ruby C. Magnetite as a precursor for green rust through the hydrogenotrophic activity of the iron-reducing bacteria Shewanella putrefaciens. Geobiology 2016, 14 (3), 237–254. 10.1111/gbi.12170. 26715461

[ref50] Miot J.; Benzerara K.; Morin G.; Bernard S.; Beyssac O.; Larquet E.; Kappler A.; Guyot F. Transformation of vivianite by anaerobic nitrate-reducing iron-oxidizing bacteria. Geobiol. 2009, 7 (3), 373–384. 10.1111/j.1472-4669.2009.00203.x. 19573166

[ref51] Hegler F.; Posth N. R.; Jiang J.; Kappler A. Physiology of phototrophic iron (II)-oxidizing bacteria: implications for modern and ancient environments. FEMS Microbiol. Ecol. 2008, 66 (2), 250–260. 10.1111/j.1574-6941.2008.00592.x. 18811650

[ref52] Luna-Zaragoza D.; Romero-Guzmán E. T.; Reyes-Gutiérrez L. R. Surface and physicochemical characterization of phosphates vivianite, Fe2(PO4)3 and hydroxyapatite, Ca5(PO4)3OH. Journal of Minerals & Materials Characterization & Engineering 2009, 8 (8), 591–609. 10.4236/jmmce.2009.88052.

[ref53] Eynard A. d.; Del Campillo M. C.; Barrón V.; Torrent J. Use of vivianite (Fe3 (PO4) 2.8 H2O) to prevent iron chlorosis in calcareous soils. Fertilizer Research 1992, 31 (1), 61–67. 10.1007/BF01064228.

[ref54] Borchert H.; Shevchenko E. V.; Robert A.; Mekis I.; Kornowski A.; Grübel G.; Weller H. Determination of nanocrystal sizes: a comparison of TEM, SAXS, and XRD studies of highly monodisperse CoPt3 particles. Langmuir 2005, 21 (5), 1931–1936. 10.1021/la0477183. 15723491

[ref55] Criscenti L. J.; Sverjensky D. A. The role of electrolyte anions (ClO 4-, NO 3-, and Cl-) in divalent metal (M 2+) adsorption on oxide and hydroxide surfaces in salt solutions. Am. J. Sci. 1999, 299 (10), 828–899. 10.2475/ajs.299.10.828.

[ref56] Leitzke T. J.; Downey J.; LaDouceur R. M.; Margrave D. M.; Wallace G. C.; Hutchins D. L. Water Treatment Method for Removal of Select Heavy Metals and Nutrient Ions Through Adsorption by Magnetite. ACS ES&T Water 2022, 2 (9), 1584–1592. 10.1021/acsestwater.2c00242.

[ref57] Dhakal P.; Matocha C.; Huggins F.; Vandiviere M. Nitrite reactivity with magnetite. Environ. Sci. Technol. 2013, 47 (12), 6206–6213. 10.1021/es304011w. 23662623

[ref58] Hotze E. M.; Phenrat T.; Lowry G. V. Nanoparticle aggregation: challenges to understanding transport and reactivity in the environment. Journal of environmental quality 2010, 39 (6), 1909–1924. 10.2134/jeq2009.0462. 21284288

[ref59] Liu W.-T. Nanoparticles and their biological and environmental applications. J. Biosci. Bioeng. 2006, 102 (1), 1–7. 10.1263/jbb.102.1. 16952829

[ref60] Baalousha M. Aggregation and disaggregation of iron oxide nanoparticles: influence of particle concentration, pH and natural organic matter. Science of the total Environment 2009, 407 (6), 2093–2101. 10.1016/j.scitotenv.2008.11.022. 19059631

[ref61] Mansor M.; Drabesch S.; Bayer T.; Van Le A.; Chauhan A.; Schmidtmann J.; Peiffer S.; Kappler A. Application of Single-Particle ICP-MS to Determine the Mass Distribution and Number Concentrations of Environmental Nanoparticles and Colloids. Environ. Sci. Technol. Lett. 2021, 8 (7), 589–595. 10.1021/acs.estlett.1c00314.

[ref62] Gorski C. A.; Nurmi J. T.; Tratnyek P. G.; Hofstetter T. B.; Scherer M. M. Redox behavior of magnetite: Implications for contaminant reduction. Environ. Sci. Technol. 2010, 44 (1), 55–60. 10.1021/es9016848. 20039733

[ref63] Milonjić S.; Kopečni M.; Ilić Z. The point of zero charge and adsorption properties of natural magnetite. Journal of Radioanalytical and Nuclear Chemistry 1983, 78 (1), 15–24. 10.1007/BF02519745.

[ref64] Anson F. C.; Barclay D. J. Anion induced adsorption of cadmium (II) on mercury from iodide and bromide media. Anal. Chem. 1968, 40 (12), 1791–1798. 10.1021/ac60268a021.

[ref65] Liu J.-F.; Zhao Z.-s.; Jiang G.-b. Coating Fe3O4 magnetic nanoparticles with humic acid for high efficient removal of heavy metals in water. Environ. Sci. Technol. 2008, 42 (18), 6949–6954. 10.1021/es800924c. 18853814

[ref66] Vermeer A. W. P.; van Riemsdijk W. H.; Koopal L. K. Adsorption of Humic Acid to Mineral Particles. 1. Specific and Electrostatic Interactions. Langmuir 1998, 14 (10), 2810–2819. 10.1021/la970624r.

[ref67] Jolsterå R.; Gunneriusson L.; Holmgren A. Surface complexation modeling of Fe3O4–H+ and Mg (II) sorption onto maghemite and magnetite. J. Colloid Interface Sci. 2012, 386 (1), 260–267. 10.1016/j.jcis.2012.07.031. 22889624

[ref68] Ho Y.; McKay G. The sorption of lead (II) ions on peat. Water research 1999, 33 (2), 578–584. 10.1016/S0043-1354(98)00207-3.

[ref69] Swindle A. L.; Cozzarelli I. M.; Elwood Madden A. S. Using Chromate to Investigate the Impact of Natural Organics on the Surface Reactivity of Nanoparticulate Magnetite. Environ. Sci. Technol. 2015, 49 (4), 2156–2162. 10.1021/es504831d. 25607467

[ref70] Hu J.-D.; Zevi Y.; Kou X.-M.; Xiao J.; Wang X.-J.; Jin Y. Effect of dissolved organic matter on the stability of magnetite nanoparticles under different pH and ionic strength conditions. Science of The Total Environment 2010, 408 (16), 3477–3489. 10.1016/j.scitotenv.2010.03.033. 20421125

