# Supplementary material for: Cu(II) and Cd(II) Removal Efficiency of Microbially Redox-Activated Magnetite Nanoparticles
Source: ACS Earth Space Chem. 2023 Oct 9;7(10):1837–47. doi: 10.1021/acsearthspacechem.2c00394 (PMC10591504; doi:10.1021/acsearthspacechem.2c00394)
Supplement: Supplementary file 1 — sp2c00394_si_001.pdf [file sp2c00394_si_001.pdf]

Cu(II) and Cd(II) removal efficiency by microbially redox-activated magnetite nanoparticles

Timm Bayer[1], Ran Wei[2], Andreas Kappler[1,3], and James M. Byrne[4,]*

[1]Geomicrobiology Group, Department of Geoscience, University of Tuebingen, Schnarrenbergstraße 94-96, 72076 Tuebingen, Germany

[2]Environmental Systems Analysis, Department of Geoscience, University of Tuebingen, Schnarrenbergstraße 94-96, 72076 Tuebingen, Germany

[3]Cluster of Excellence: EXC 2124: Controlling Microbes to Fight Infection, 72074 Tuebingen, Germany

[4]School of Earth Sciences, University of Bristol, Wills Memorial Building, Queens Road BS8 1RJ, Bristol, United Kingdom.

SUPPORTING INFORMATION

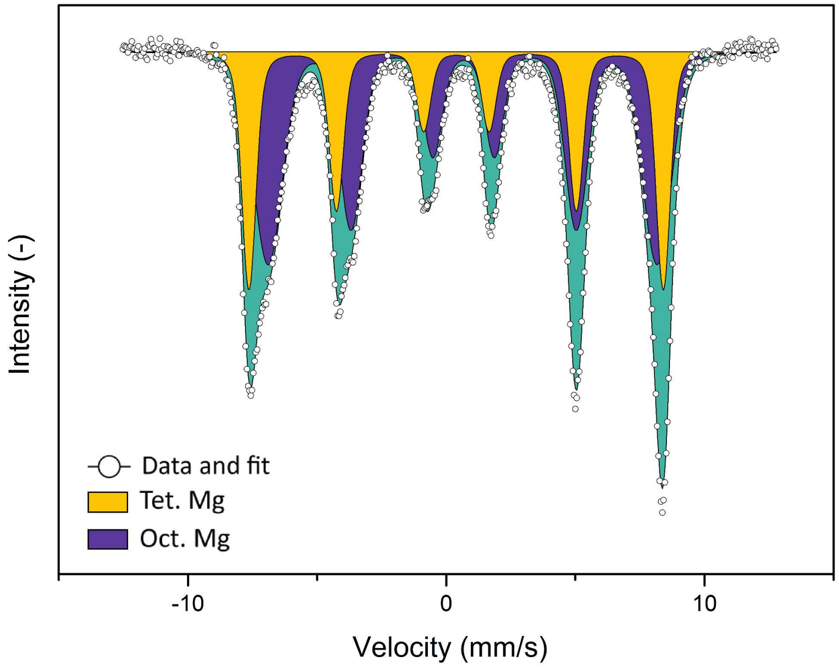

Figure S1: Mössbauer spectrum of unmodified magnetite before start of experiments, collected at 140 K. Circles correspond to raw data. Yellow sextet Fe in magnetite in tetrahedral coordination, and purple sextet Fe in magnetite in octahedral coordination.

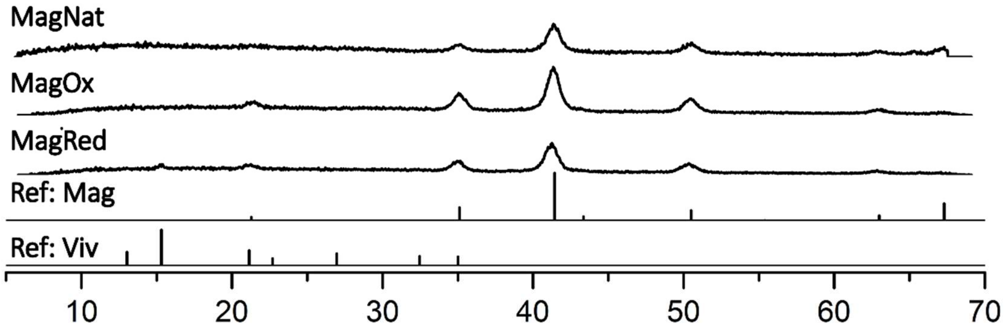

Figure S2: μXRD patterns collected from microbially oxidized and microbially reduced magnetite. References shown for magnetite and vivianite (Ref: Mag and Ref: Viv).

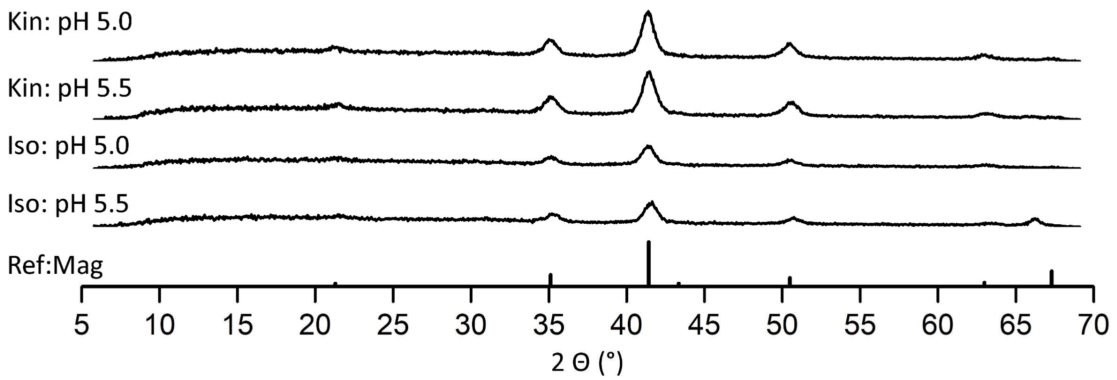

Figure S3: μXRD patterns collected of native magnetite and Cu^{2+}: kinetic experiments (Kin) at pH 5.0 and pH 5.5 and isotherm experiments (Iso) at pH values 5.0, 5.5, 6.5, and 7.3. Bottom bars show reference for magnetite (Ref: Mag).

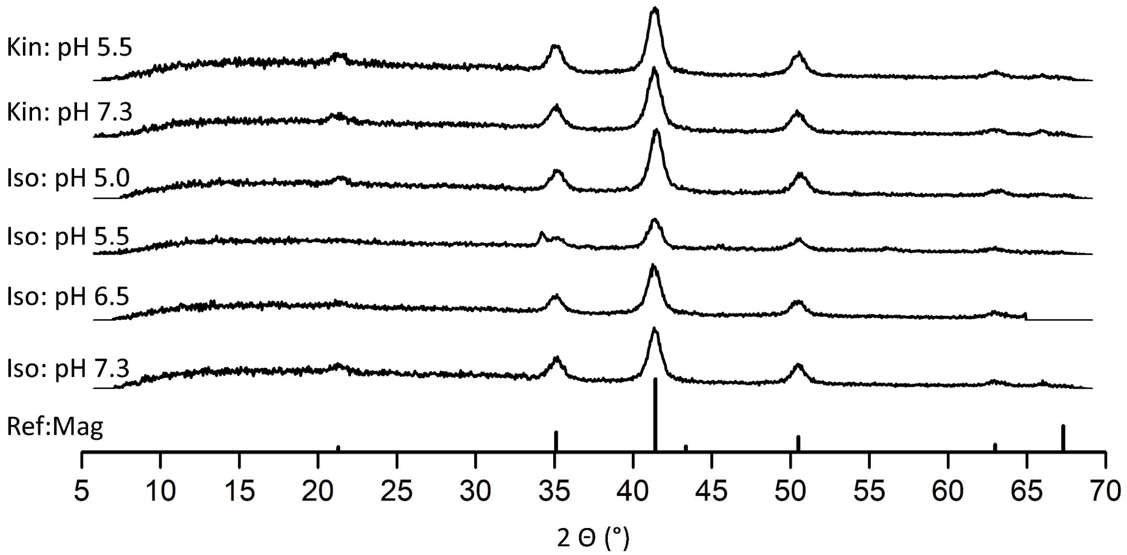

Figure S4: μXRD patterns collected of native magnetite and Cd^{2+}. Kinetic experiments (Kin) at pH 5.5 and pH 7.3 and Isotherm experiments (Iso) at pH values 5.0, 5.5, 6.5, and 7.3 Bottom bars show reference for magnetite (Ref: Mag).

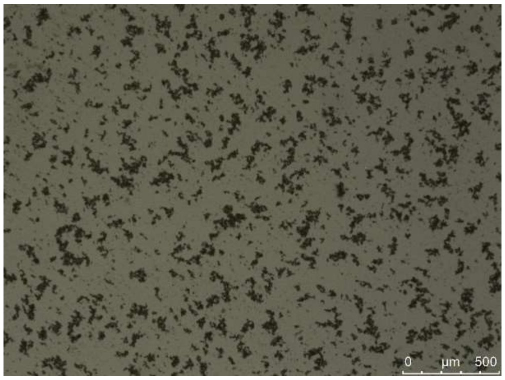

Figure S5: Washed MNPs (overlayed image) - After oxidation MNPs were washed with anoxic $NaNO_3$ five times.

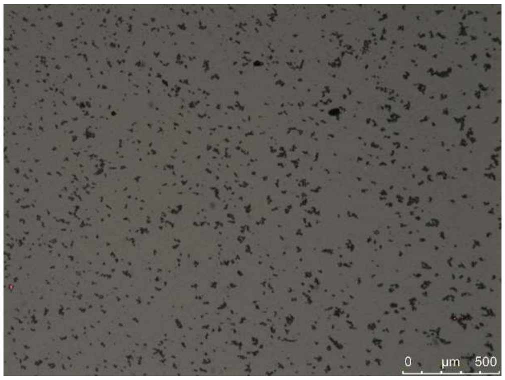

Figure S6: Washed MNPs (overlayed image) - After reduction MNPs were washed with anoxic $NaNO_3$ five times.

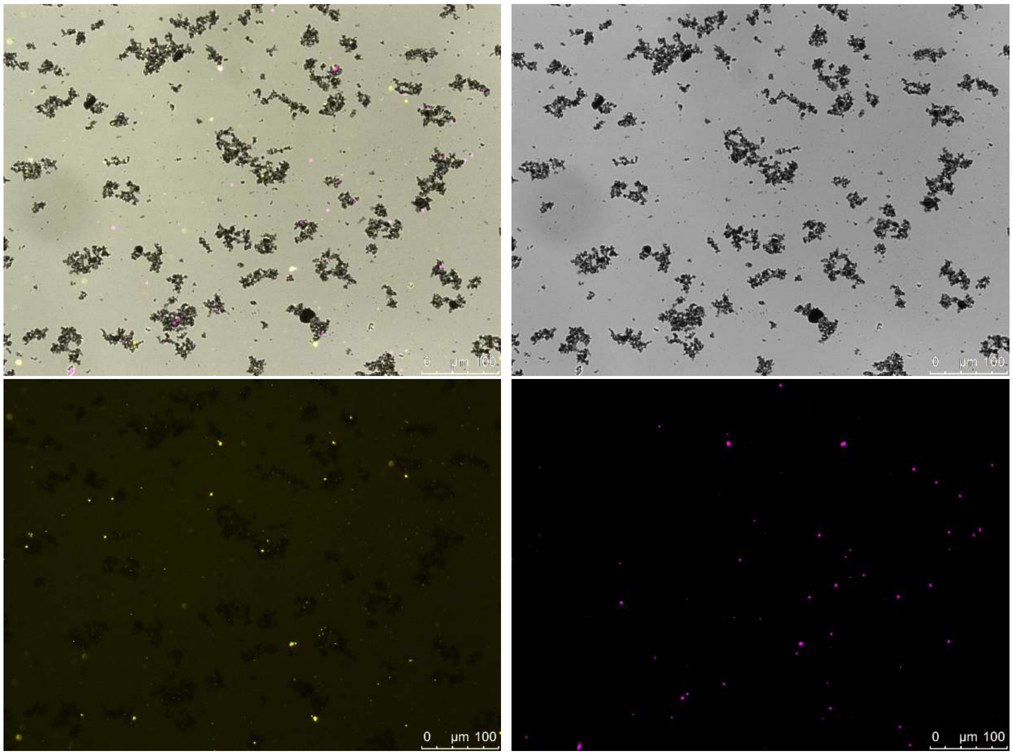

Figure S7: Partially washed MNPs - After reduction MNPs were washed with $NaNO_3$ once. Colours indicate presence of bacteria (top left - Overlay, top right – brightfield, bottom left – living cells, bottom right – dead cells).

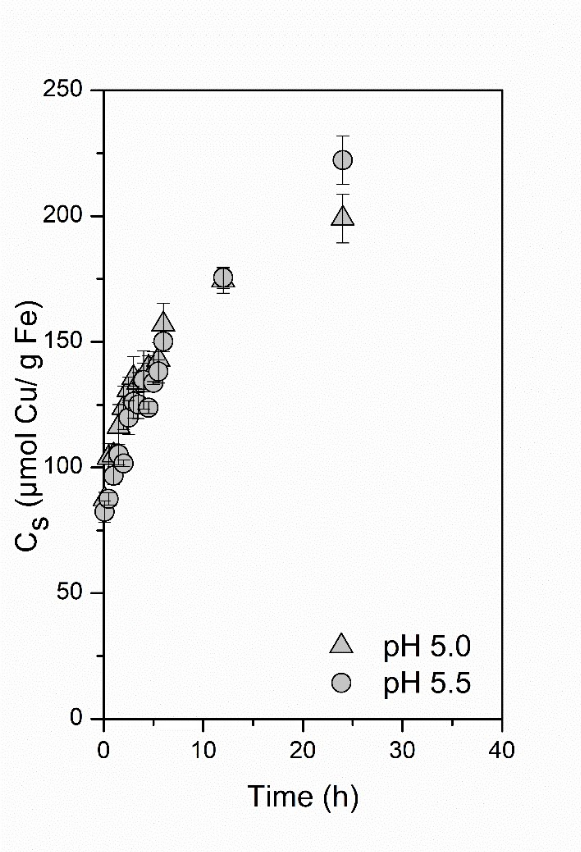

Figure S8: Kinetic behaviour of Cu^{2+} adsorption on magnetite nanoparticles at pH 5.0 (triangles) and pH 5.5 (circles) with native MNPs (grey). Triplicate bottles were incubated with magnetite (9 mM Fe) and 750 µM Cu^{2+}. Adsorbed Cu (µmol) on mass of magnetite (as g Fe) was regularly determined via MP-AES.

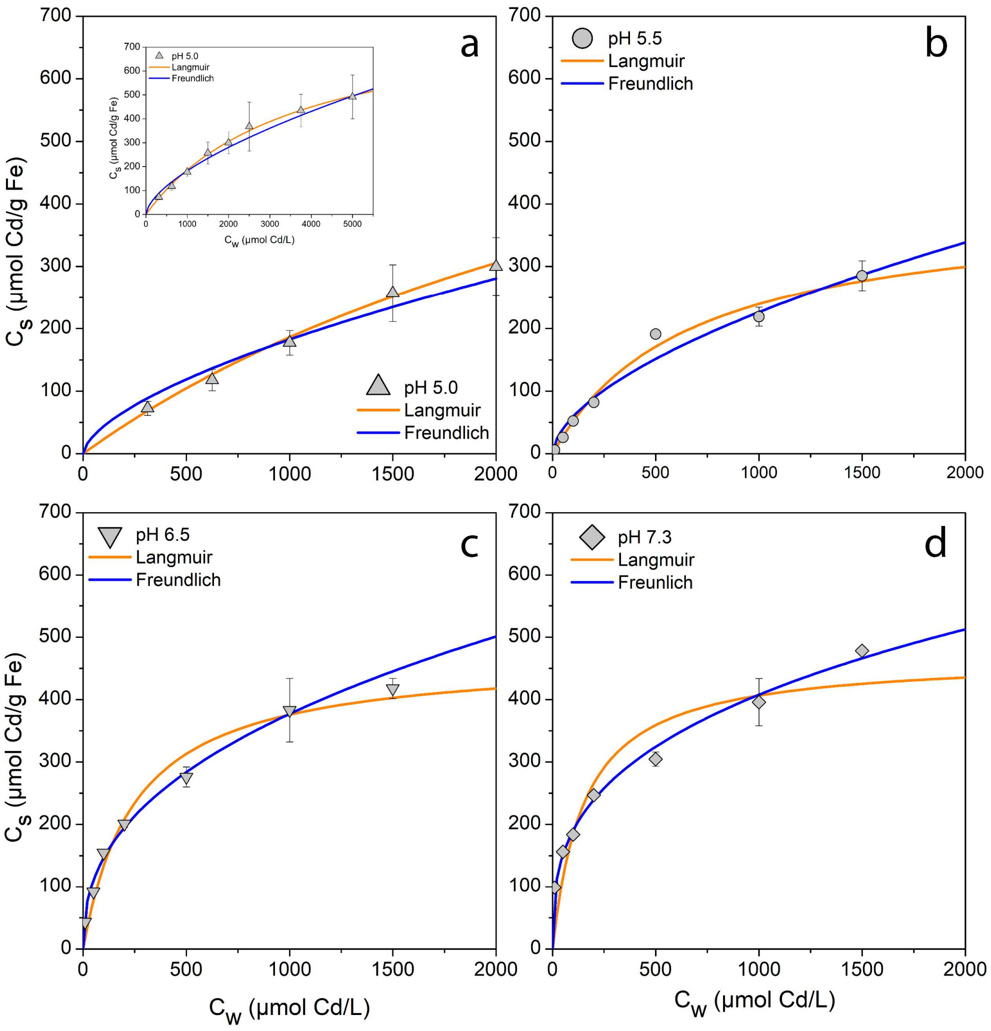

Figure S9: Measured data and fit isotherms for Cd^{2+} pH 5.0 (pyramids, panel a), 5.5 (circles panel b), 6.5 (triangles, panel c) and 7.3 (diamonds, panel d) with native (grey). Triplicate bottles with increasing Cd^{2+} concentrations were incubated for 24h and the amount of adsorbed Cu (in µmol) on mass of magnetite (as g Fe) was determined via MP-AES. Langmuir (orange) and Freundlich (blue) isotherms were fit to the data. The inset for pH 5.0 in panel a shows the entire range of the performed isotherm.

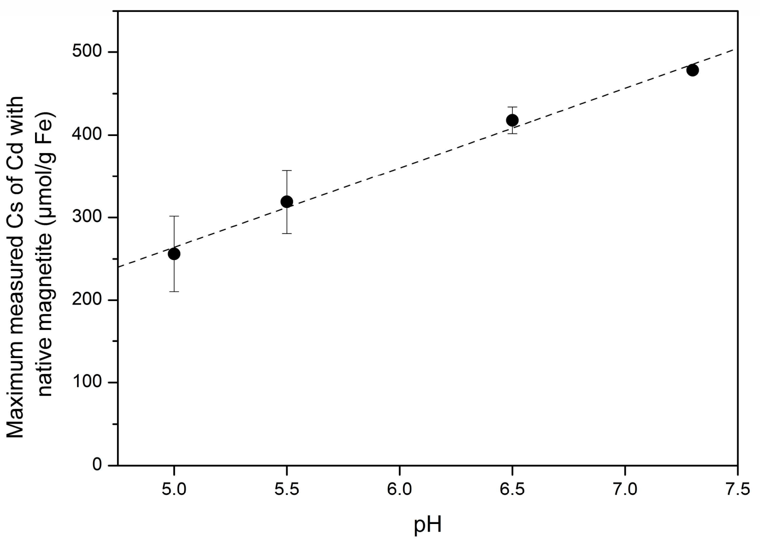

Figure S10: Linear relationship between increased pH of performed isotherms and maximum adsorbed Cd^{2+} for experiments performed with native magnetite.

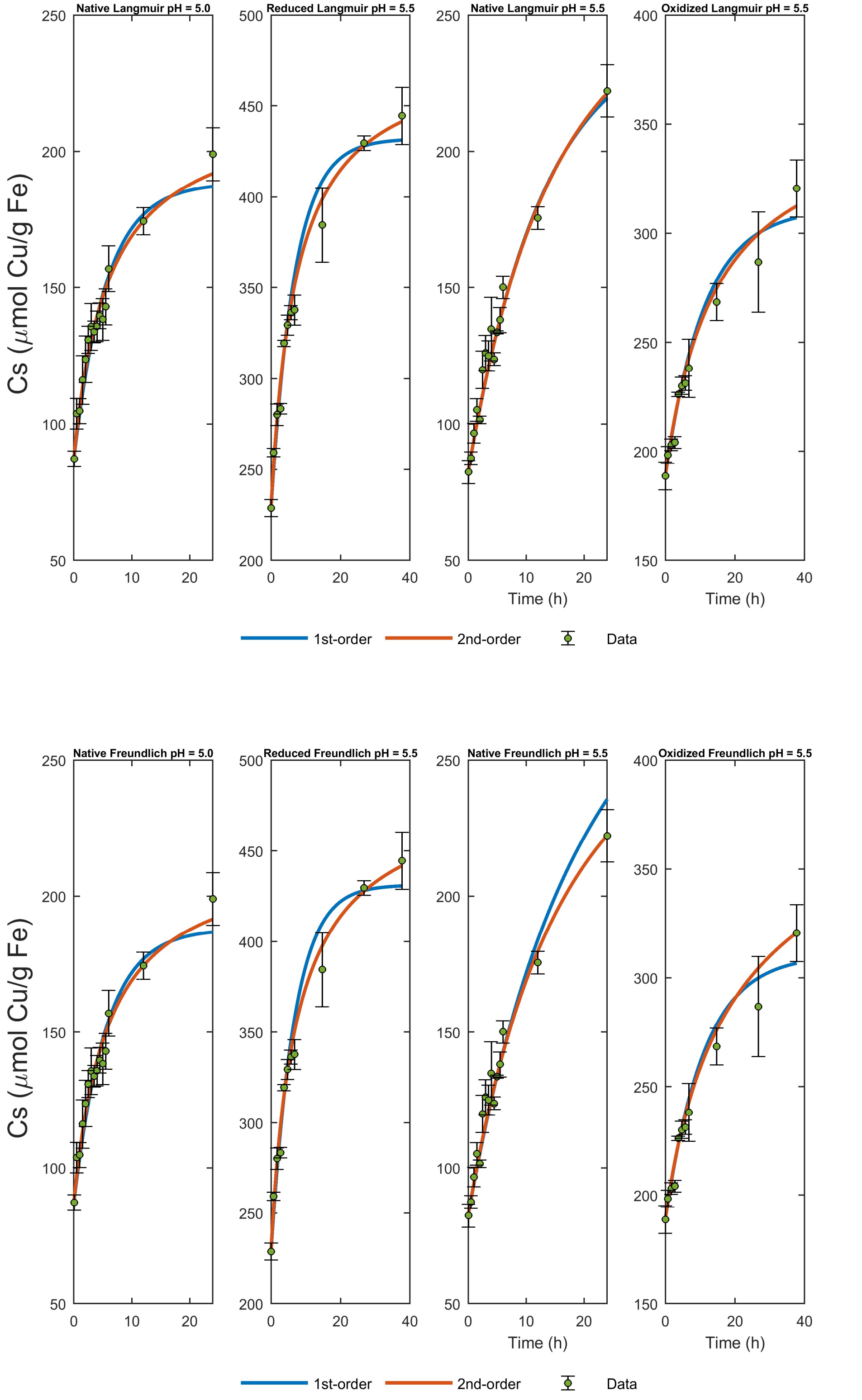

Figure S11: Data and fit model of kinetic experiments for Cu at pH 5.5 with first and second order (blue and orange) kinetics parameter derived from either Langmuir or Freundlich isotherms for equilibrium concentration.

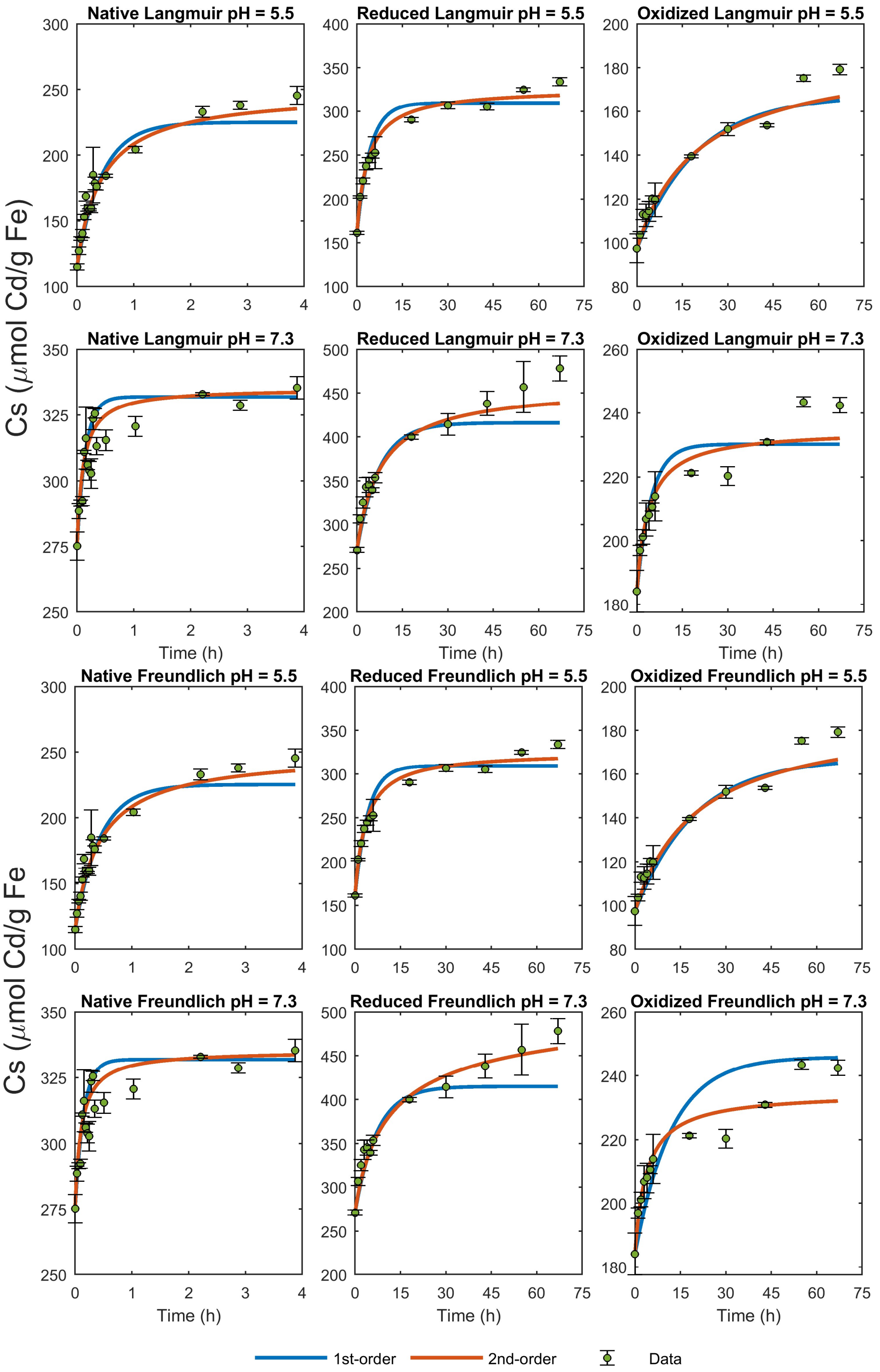

Figure S12: Data and fit model of kinetic experiments for Cu at pH 5.5 and 7.3 with first and second order (blue and orange) kinetics parameter with either Langmuir or Freundlich isotherm for equilibrium concentration.

Table S1: Fitting results of Mössbauer spectroscopy. δ – isomer shift; ΔE_Q – quadrupole splitting; B_{hf} – hyperfine magnetic field; stdev(B_{hf}) – standard deviation of hyperfine magnetic field; R.A. – Relative abundance; red. $\chi 2$ – goodness of fit.

	δ (mm/s)	ΔE_Q (mm/s)	B_{hf} (T)	stdev (B_{hf}) (T)	R.A. (%)	Error	Red. $\chi 2$
Sextet 1	0.655	-0.042	46.725	2.52547	62.6	1.4	1.49
Sextet 2	0.388	-0.011	49.756	3.87E-07	37.4	1.4	

Table S2: Fitting parameters for magnetite isotherms collected with Cd^{2+} and Cu^{2+} contacted with native, reduced, and oxidized magnetite at different pH values. Upper part showing Freundlich fitting parameters and lower part showing Langmuir fitting parameters. Fits with * at pH 5.0 indicate that a narrower range of values were used to fit the range of the other pH values.

Isotherms fit to Freundlich model

Magnetite	Element	pH	k ((µmol g^{-1})(L g^{-1})^n)	SD	rel. err.	n	SD	rel. err.	NRMSE
native	Cd	5.00	2.52	0.61	1.84	0.62	0.12	1.13	0.0460
	Cd	5.50	4.12	0.75	2.11	0.58	0.12	1.20	0.0603
	Cd	6.50	22.20	0.25	1.29	0.41	0.09	1.10	0.3454
	Cd	7.30	41.72	0.17	1.18	0.33	0.08	1.08	0.0293
reduced	Cd	5.50	28.28	0.68	1.98	0.33	0.31	1.37	0.0790
oxidized	Cd	5.50	20.75	0.25	1.29	0.34	0.11	1.12	0.0333
reduced	Cd	7.30	62.24	0.29	1.33	0.32	0.14	1.15	0.0396
oxidized	Cd	7.30	64.90	0.16	1.17	0.23	0.10	1.11	0.0322
native*	Cd	5*	0.51	0.27	1.31	0.85	0.05	1.05	0.1995
native	Cu	5.00	23.81	0.18	1.19	0.32	0.07	1.07	0.0303
native	Cu	5.50	28.62	0.18	1.20	0.31	0.09	1.09	0.0368
reduced	Cu	5.50	50.02	0.48	1.62	0.33	0.22	1.25	0.0790
oxidized	Cu	5.50	27.66	0.41	1.50	0.33	0.19	1.21	0.0709
native*	Cu	5.0*	32.30	0.07	1.07	0.27	0.04	1.04	0.0196

Isotherms fit to Langmuir model

Magnetite	Element	pH	q_{max} (µmol g^{-1})	SD	rel. err.	k_{ads} (µmol L^{-1})	SD	rel. err.	NRMSE
native	Cd	5.00	851.2	0.1	1.1	3572.6	0.2	1.2	0.0120
	Cd	5.50	399.1	0.2	1.2	666.6	0.4	1.5	0.0426
	Cd	6.50	470.1	0.1	1.1	251.1	0.3	1.4	0.0549
	Cd	7.30	468.4	0.2	1.2	152.4	0.6	1.9	0.1125
reduced	Cd	5.50	317.6	0.1	1.3	131.6	0.2	1.3	0.0276
oxidized	Cd	5.50	254.2	0.2	2.1	177.6	0.8	2.1	0.1043
reduced	Cd	7.30	663.7	0.2	1.9	149.6	0.6	1.9	0.0864
oxidized	Cd	7.30	339.7	0.2	2.4	66.9	0.9	2.4	0.1407
native*	Cd	5*	1231.6	0.4	1.5	5761.9	0.5	1.7	0.2973
native	Cu	5.00	375.0	0.11	1.11	692.10	0.37	1.45	0.0903
native	Cu	5.50	297.1	0.08	1.08	195.66	0.31	1.36	0.0747
reduced	Cu	5.50	561.7	0.04	1.04	131.60	0.16	1.18	0.0276
oxidized	Cu	5.50	308.8	0.03	1.04	138.32	0.13	1.14	0.0233
native*	Cu	5.0*	262.4	0.03	1.03	247.71	0.13	1.14	0.4409

Table S3: Fitting parameters for magnetite kinetic experiments Cu^{2+} contacted with native, reduced, and oxidized magnetite at different pH values.

Kinetics fit to Langmuir model							
Magnetite	Element	pH	$k_{sorb,1}$ (s^{-1}) or $k_{sorb,2}$ (μmol^{-1} g s^{-1})	order	q_{max} (μmol g^{-1})	k_{ads} (μmol L^{-1})	NRMSE
native	Cu	5.0	5.00e-05	first	188.53	0.0006924232	0.0559
native	Cu	5.5	6.32e-06	first	4301582.11	5119248.0615	0.0428
reduced	Cu	5.5	4.08e-05	first	432.03	0.0001317265	0.0541
oxidized	Cu	5.5	2.47e-05	first	311.43	0.0001387612	0.0596
native	Cu	5.0	5.20e-08	second	7517257.53	13173464.532	0.0443
native	Cu	5.5	9.17e-09	second	1175.49	507.42192	0.0421
reduced	Cu	5.5	1.51e-07	second	482.23	0.0001317433	0.0372
oxidized	Cu	5.5	4.74e-09	second	640723.00	472627.2032	0.0504

Kinetics fit to Freundlich model							
Magnetite	Element	pH	$k_{sorb,1}$ (s^{-1}) or $k_{sorb,2}$ (μmol^{-1} g s^{-1})	order	k ((μmol g^{-1})(L g^{-1})^n)	n	NRMSE
native	Cu	5.0	3.10e-05	first	4.76	0.60	0.0566
native	Cu	5.5	3.88e-08	first	2.86	1.56	0.0512
reduced	Cu	5.5	6.66e-06	first	5.00	0.86	0.0561
oxidized	Cu	5.5	9.67e-06	first	2.77	0.79	0.0602
native	Cu	5.0	2.43e-07	second	71.43	0.19	0.0449
native	Cu	5.5	1.04e-08	second	35.80	0.44	0.0417
reduced	Cu	5.5	1.39e-13	second	163.24	1.12	0.0561
oxidized	Cu	5.5	1.45e-13	second	2.77	1.65	0.0551

Table S4: Fitting parameters for magnetite kinetic experiments Cd^{2+} with Freundlich equilibrium, contacted with native, reduced, and oxidized magnetite at different pH values.

Kinetics fit to Freundlich model							
Magnetite	Element	pH	$k_{sorb,1}$ (s^{-1}) or $k_{sorb,2}$ (μmol^{-1} g s^{-1})	order	k ((μmol g^{-1})(L g^{-1})^n)	n	NRMSE
native	Cd	7.3	1.39e-03	first	75.90	0.23	0.1473
native	Cd	5.5	2.47e-4	first	0.31	1.08	0.0795
reduced	Cd	5.5	5.83e-05	first	210.44	0.07	0.0744
reduced	Cd	7.3	3.37e-05	first	285.12	0.06	0.1224
oxidized	Cd	5.5	9.04e-06	first	2.08	0.70	0.0856
oxidized	Cd	7.3	1.39e-05	first	6.49	0.57	0.1569
native	Cd	7.3	1.67e-05	second	27.60	0.39	0.1206
native	Cd	5.5	4.51e-07	second	0.31	1.13	0.0595
reduced	Cd	5.5	3.68e-07	second	223.40	0.06	0.0462
reduced	Cd	7.3	1.39e-13	second	6.22	1.58	0.0594
oxidized	Cd	5.5	1.35e-07	second	123.42	0.07	0.0733
oxidized	Cd	7.3	1.30e-06	second	174.13	0.05	0.0927

Table S5: Fitting parameters for magnetite kinetic experiments Cd^{2+} with Langmuir equilibrium, contacted with native, reduced, and oxidized magnetite at different pH values.

Kinetics fit to Langmuir model							
Magnetite	Element	pH	$k_{sorb,1}$ (s^{-1}) or $k_{sorb,2}$ (μmol^{-1} g s^{-1})	order	q_{max} (μmol L^{-1})	k_{ads} (μmol L^{-1})	NRMSE
native	Cd	7.3	7.23e-04	first	80048645.81	142458828.1852	0.1468
native	Cd	5.5	2.64e-04	first	468902468.63	919212790.2676	0.0800
reduced	Cd	5.5	6.5e-05	first	309.62	0.0001316016	0.0735
reduced	Cd	7.3	3.86e-05	first	416.00	0.000149647	0.1201
oxidized	Cd	5.5	1.41e-05	first	167.28	0.0001795486	0.0853
oxidized	Cd	7.3	5.93e-05	first	230.23	7.023212e-05	0.1125
native	Cd	7.3	1.62e-05	second	591.33	444.7175	0.1210
native	Cd	5.5	5.91e-07	second	19999474.62	30140634.7864	0.0601
reduced	Cd	5.5	4.69e-07	second	326.69	0.0001316818	0.0448
reduced	Cd	7.3	1.94e-07	second	457.55	0.0001496525	0.0784

Table S6: Summary of properties of MNPs

Type	Fe(II)/Fe(III)	Open circuit potential E_{OCP} [mV][1]	*Average crystal size d [nm]*[2]	BET SSA [m^2 g^{-1}]	Calculated SSA from *d* [m^2 g^{-1}][3]
oxidized	0.26 ± 0.02	-0.12	10.23		101.0
native	0.42 ± 0.01	-0.36	10.29 (12.49*)	92.73	100.4
reduced	0.54 ± 0.03	-0.54	9.59		107.7

*calculated from BET measurement[3]

References

(1) Gorski, C. A.; Nurmi, J. T.; Tratnyek, P. G.; Hofstetter, T. B.; Scherer, M. M. Redox behavior of magnetite: Implications for contaminant reduction. *Environmental Science & Technology* **2010**, *44* (1), 55-60.

(2) Patterson, A. L. The Scherrer Formula for X-Ray Particle Size Determination. *Physical Review* **1939**, *56* (10), 978-982. DOI: 10.1103/PhysRev.56.978.

(3) Etique, M.; Jorand, F. P.; Ruby, C. Magnetite as a precursor for green rust through the hydrogenotrophic activity of the iron-reducing bacteria Shewanella putrefaciens. *Geobiology* **2016**, *14* (3), 237-254.
